# Supplementary material for: Functional implications of the conformational landscape of a multidrug transporter revealed by Zebrafish Abcb4 structures
Source: Nat Commun. 2026 May 30;17:7014. doi: 10.1038/s41467-026-73751-4 (PMC13392234; doi:10.1038/s41467-026-73751-4)
Supplement: Supplementary file 1 — Supplementary Information [file 41467_2026_73751_MOESM1_ESM.pdf]

Functional Implications of the Conformational Landscape of a Multidrug Transporter Revealed by Zebrafish Abcb4 Structures

Jingyu Zhan[1], Chao-Ming Hsieh[1], Lothar Esser[1], Zabrina C. Lang[1,2], Abraham J Morton[1,2], Robert Robey[1], Fei Zhou[1], Suresh V. Ambudkar[1], Rick K. Huang[1,2], Michael M. Gottesman[1], and Di Xia[1*]

[1]Laboratory of Cell Biology, National Cancer Institute
[2]NIH Intramural Cryo-EM Consortium
National Institutes of Health, Bethesda, MD, USA 20892

*Corresponding author. Email: xiad@mail.nih.gov

Supplemental Information

Supplementary Table 1

Supplementary Figures 1 to 11

Other Supplementary Material for this manuscript includes the following:

Supplementary Movies 1 to 2

Table S1 | Cryo-EM data collection, model refinement and validation statistics

	Abcb4/Apo			Abcb4/Tariquidar			Abcb4/Elacridar			Abcb4/Vincristine		
**Data collection**[a]												
Total no. of micrographs		3,764			12,281			12,136			3,427	
**Data processing**												
Reconstruction software		CryoSparc			CryoSparc/Relion			CryoSparc/Relion			CryoSparc	
No. extracted particles		1,286,409			6,064,148			5,253,173			1,841,052	
No. selected 2D particles		880,810			2,359,321			2,498,124			540,770	
Conformation designation[b]	IF^{Nar}	IF^{Med}	IF^{Wide}	TQR-IF^{Nar}	TQR-IF^{Med}	TQR-IF^{Wide}	ELA-IF^{Nar}	ELA-IF^{Med}	ELA-IF^{Wide}	VCR-IF^{Nar}	VCR-IF^{Med}	VCR-IF^{Wide}
No. particles for final map	180,825	190,766	138,295	581,887	445,705	321,047	478,434	463,553	431,828	110,004	129,591	101,031
Map resolution (Å)[c]	3.29	3.39	3.59	3.02	3.18	3.54	3.00	3.16	3.31	3.95	3.70	4.09
Map sharpening B-factor ($Å^2$)	-116.0	-116.9	-114.6	-126.2	-110.1	-129.9	-109.9	-116.9	-110.9	-155.5	-129.2	-178.1
**PHENIX refinement statistics**												
Initial model used	PDB:5KO2	PDB:5KO2	PDB:5KO2	ModelAngelo	ModelAngelo	ModelAngelo	ModelAngelo	ModelAngelo	ModelAngelo	ModelAngelo	ModelAngelo	ModelAngelo
Model composition												
Non-hydrogen Atoms	9,010	9,010	8,978	9,322	9,267	9,001	9,273	9,044	8,984	9,243	9,034	9,013
No. of protein residues	1,154	1,154	1,150	1,178	1,176	1,152	1,179	1,159	1,151	1,176	1,157	1,155
No. of Ligands	Mg^{2+}: 2<br>ATPγS: 2	Mg^{2+}: 2<br>ATPγS: 2	Mg^{2+}: 2<br>ATPγS: 2	Mg^{2+}: 2<br>ATPγS: 2<br>TQR: 2	Mg^{2+}: 2<br>ATPγS: 2<br>TQR: 1<br>CLR[e]: 1	Mg^{2+}: 2<br>ATPγS: 2	Mg^{2+}: 2<br>ATPγS: 2<br>ELA: 1<br>CHD[e]:1	Mg^{2+}: 2<br>ATPγS: 2	Mg^{2+}: 2<br>ATPγS: 2	Mg^{2+}: 2<br>ATPγS: 2<br>VCR: 1	Mg^{2+}: 2<br>ATPγS: 2	Mg^{2+}: 2<br>ATPγS: 2
Overall B-factor ($Å^2$)												
Protein	67.79	68.48	97.68	54.30	61.88	73.48	112.4	107.5	137.4	205.8	167.5	260.9
Ligands	102.81	98.52	124.02	70.43	85.66	95.13	133.6	135.9	160.7	260.1	196.6	290.6
RMS deviations												
Bond length (Å)	0.005	0.003	0.005	0.003	0.004	0.005	0.003	0.003	0.003	0.007	0.003	0.004
Bond angle (°)	0.663	0.554	1.068	0.677	0.965	0.977	0.681	0.657	0.598	1.229	0.672	0.964
Model resolution[d] (Å)	3.72	3.77	3.99	3.28	3.36	3.84	3.28	3.38	3.54	4.31	4.03	4.54
Real-space correlation												
CC (mask)	0.80	0.79	0.79	0.83	0.85	0.74	0.88	0.85	0.82	0.87	0.83	0.85
CC (volume)	0.79	0.78	0.78	0.82	0.84	0.73	0.87	0.84	0.81	0.87	0.83	0.85
CC (peaks)	0.66	0.66	0.58	0.72	0.72	0.58	0.64	0.60	0.52	0.58	0.52	0.53
**Validation**												
MolProbity score	2.05	2.33	2.40	2.20	2.36	1.99	2.02	1.57	1.96	2.64	1.69	1.80
Clash score	5.89	8.09	10.39	9.57	8.85	4.53	4.81	3.35	8.95	20.15	4.89	7.98
Rotamer outliers (%)	2.62	3.04	2.73	2.86	4.10	3.05	3.98	1.67	1.58	4.00	1.25	0.00
Ramachandran plot												
Favored (%)	93.82	90.94	90.56	95.05	93.68	94.07	95.48	96.01	95.28	94.44	94.70	94.69
Allowed (%)	5.92	8.71	9.00	4.78	6.15	5.76	4.43	3.90	4.72	5.56	5.21	5.22
Disallowed (%)	0.26	0.35	0.44	0.17	0.17	0.17	0.09	0.09	0.00	0.00	0.09	0.09
**Data deposition**												
PDB	9ODY	9OE0	9ODZ	9OE1	9OE2	9OE3	9OE4	9OE5	9OE6	9OE7	9OE8	9OE9
EMD	EMD-70380	EMD-70382	EMD-70381	EMD-70383	EMD-70384	EMD-70385	EMD-70386	EMD-70387	EMD-70388	EMD-70389	EMD-70390	EMD-70391

a - All data sets were collected on a Thermo Fisher Scientific Titan Krios microscope (accelerating voltage of 300kV) equipped with a Gatan K3 camera at a nominal magnification of 105,000x and a pixel size of 0.415 Å (0.83Å binned). Each movie was dose fractionated into 50 frames with a total electron exposure of 54.4 $e^-/Å^2$ at a defocus range of 0.7-2.0 μm.

b - Conformation designation: Nar - narrow separation between two NBDs; Med - medium separation; Wide - wide separation.

c - map resolution was based on FSC threshold of 0.143.

d - model resolution is defined as the FSC of 0.5 for model vs. map

e - CLR, cholesterol; CHD, cholate.

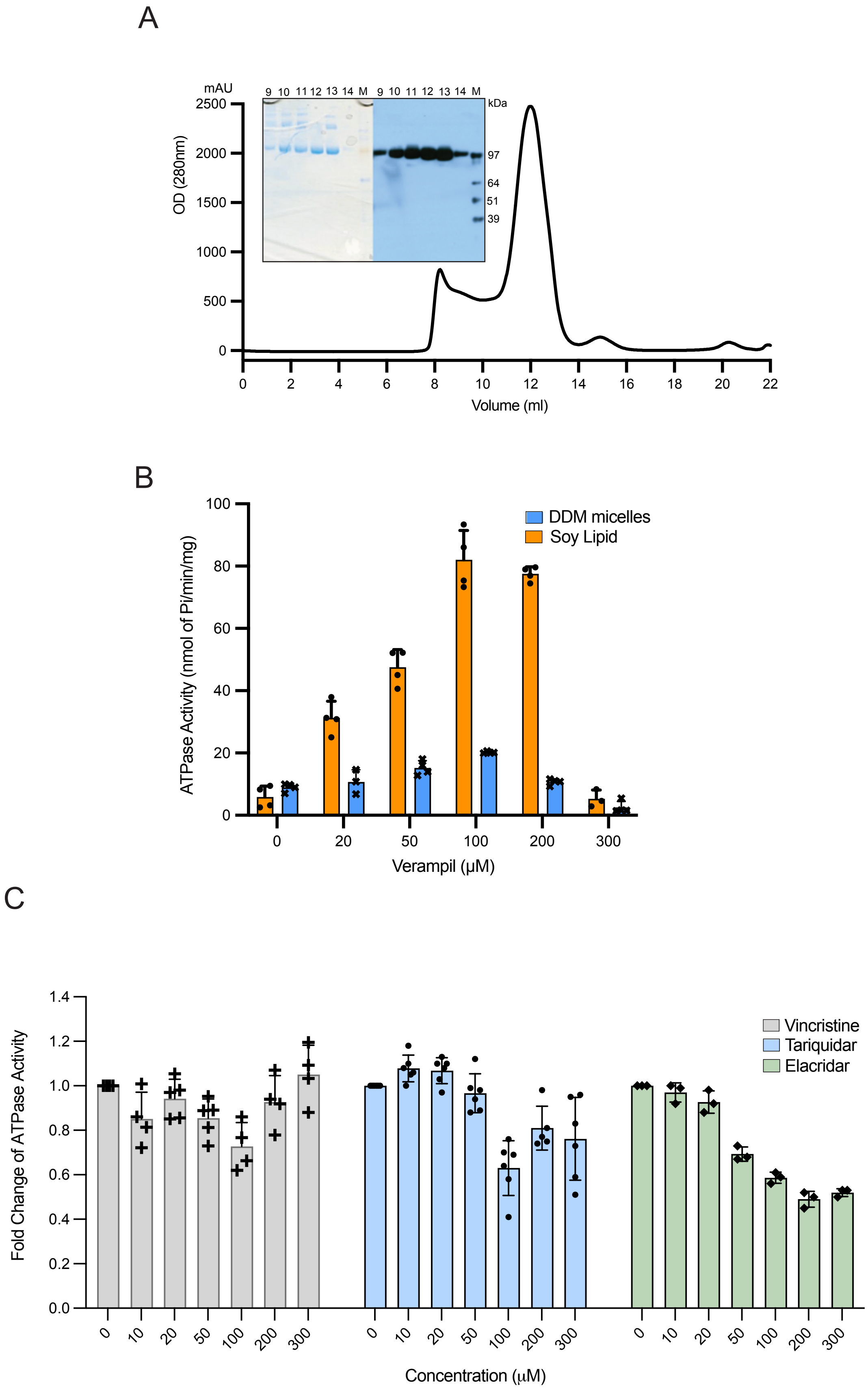

Zhan et al., Figure S1

Figure S1. Expression, purification, biochemical characterization of recombinant DrAbcb4. (A) Size-exclusion chromatogram (SEC) of isolated DrAbcb4 using a Superdex™ 200 Increase 10/300 GL column. Inset: SDS-PAGE (left) and Western Blot (right) analyses of SEC peak fractions showing monodispersity of purified sample and cross-reaction of the monoclonal antibody C219 with DrAbcb4. Uncropped scans of the SDS-PAGE and Western blot are provided in the Source Data file. (B) Basal and verapamil-modulated ATPase activity in detergent and in soy lipid bicelle solutions. Data are presented as mean ± SD from at least three independent experiments (n ≥ 3). Source data are provided. (C) Concentration-dependent ATPase activity of DrAbcb4 in the presence of vincristine, tariquidar or elacridar. Data shown are mean ± SD of at least three independent experiments (n ≥ 3). Source data are provided.

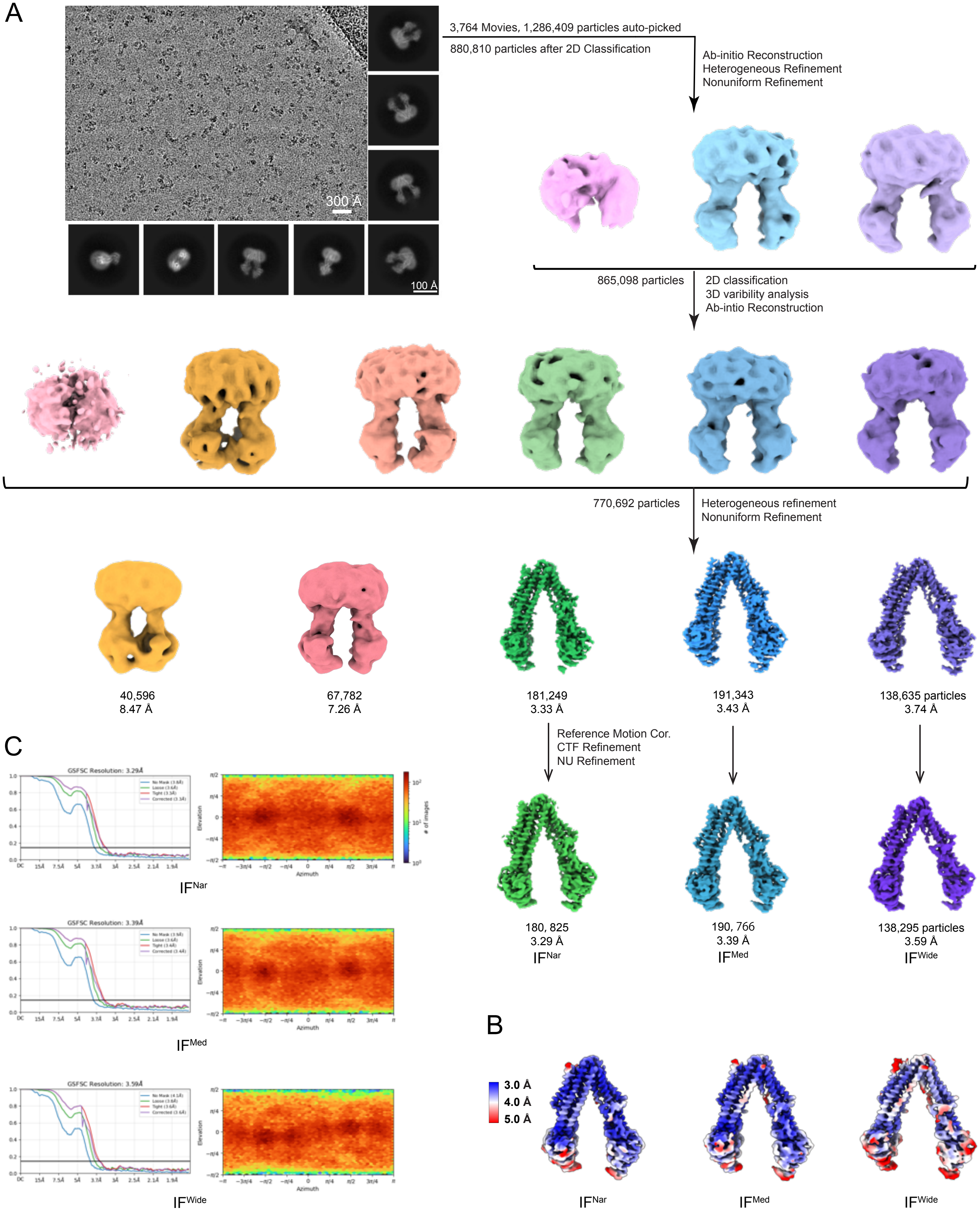

Zhan et al., Figure S2

Figure S2. Cryo-EM data processing for DrAbcb4 in the presence of ATPγS/Mg^{2+}. (A) Processing workflow included particle picking, 2D classification, 3D reconstruction and classification that ultimately led to three high-resolution structures of DrAbcb4 in IF conformations. (B) Local resolution maps for the three final structures. (C) Gold-standard Fourier shell correlation (FSC) curves (left) and viewing direction distribution plots (right) for the three reconstructions.

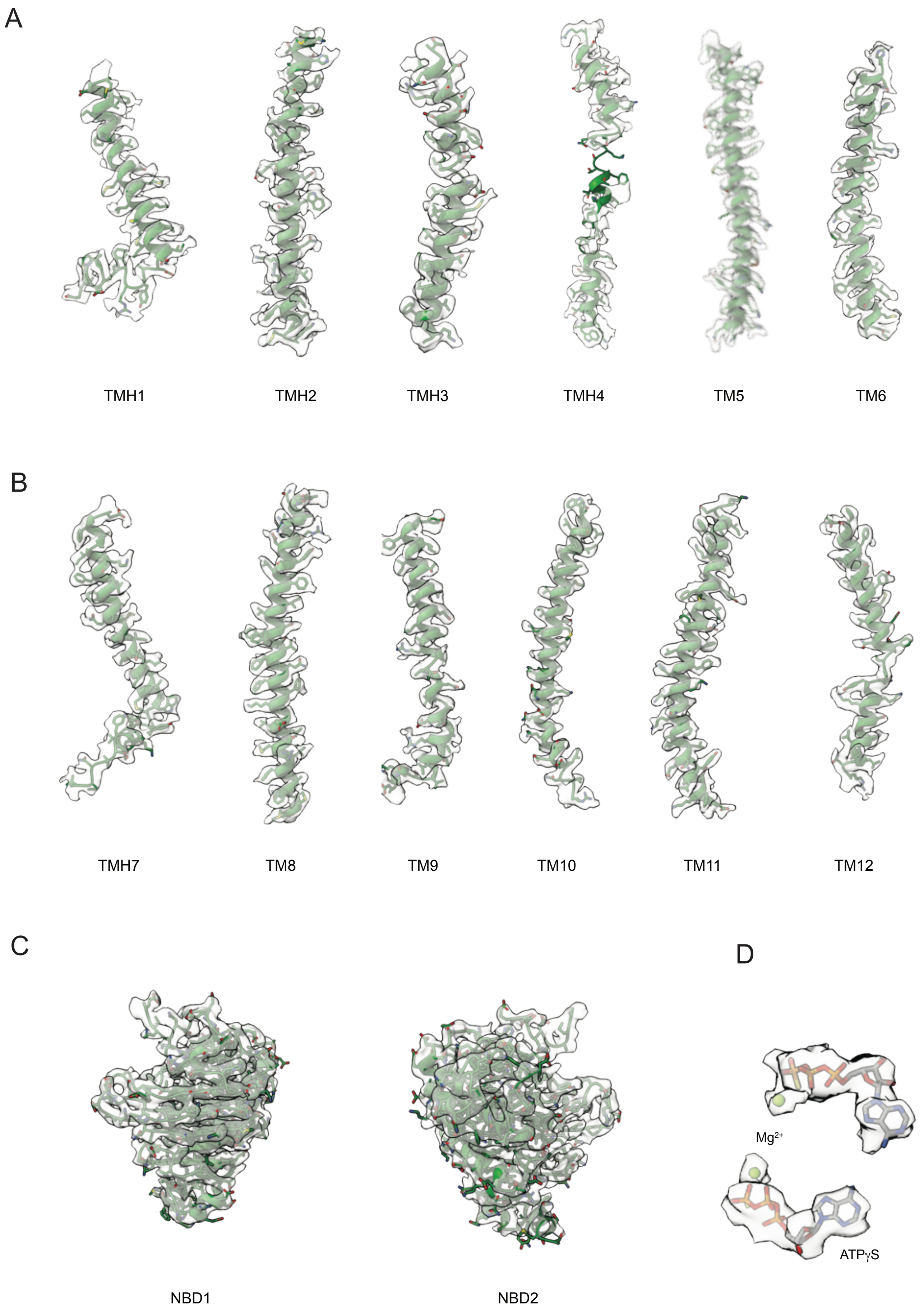

Zhan et al., Figure S3

Figure S3. Representative EM density for various regions of DrAbcb4. Selected regions of the sharpened EM density map of DrAbcb4 in the Apo IF^{Nar} conformation are shown as semi-transparent surfaces contoured at a level of 0.018, overlaid with the corresponding atomic models. Displayed regions are: (A) TMHs 1-6, (B) TMHs 7-12, (C) NBD1 and NBD2, and (D) bound ATPγS.

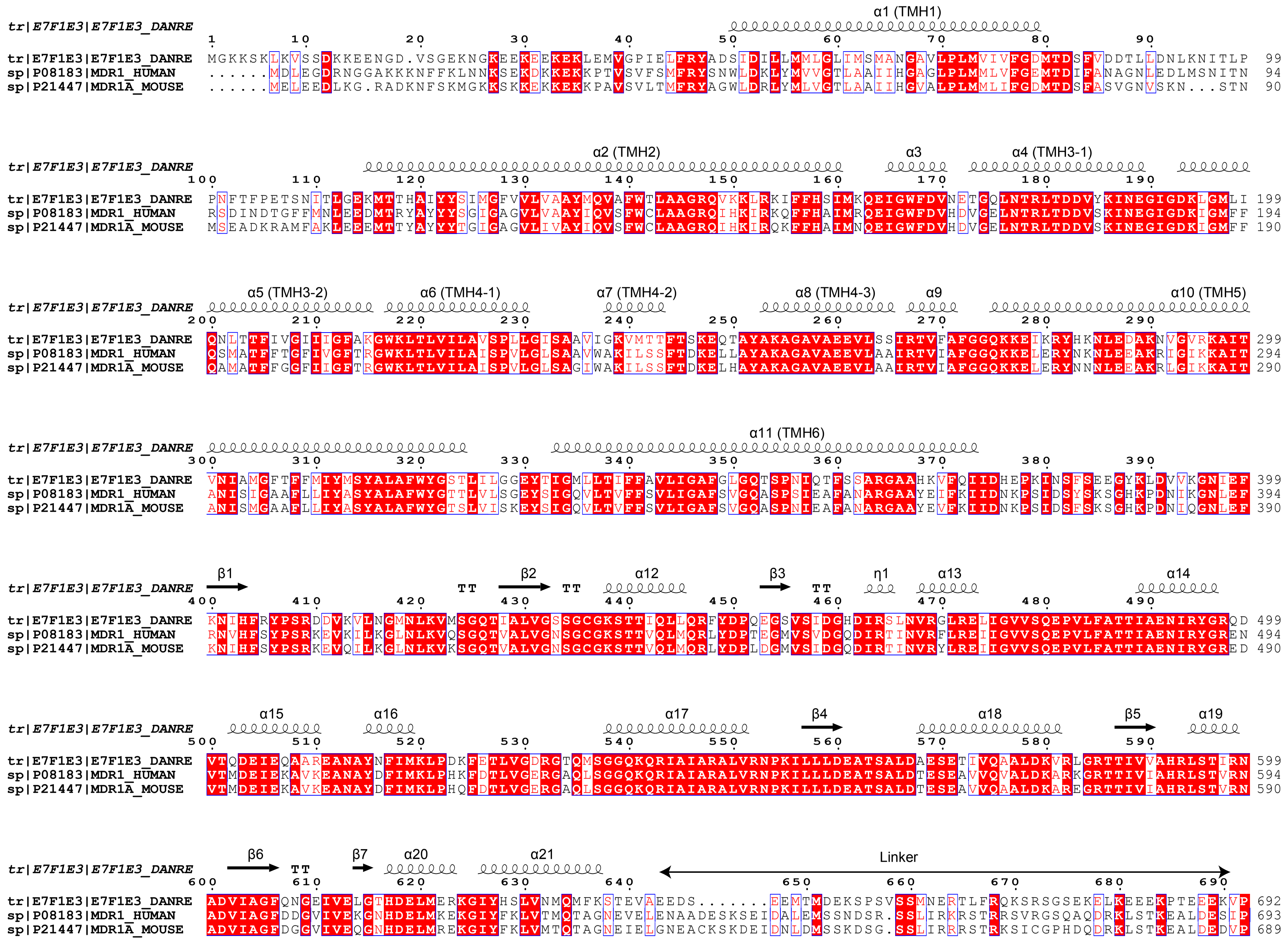

Zhan et al., Figure S4A

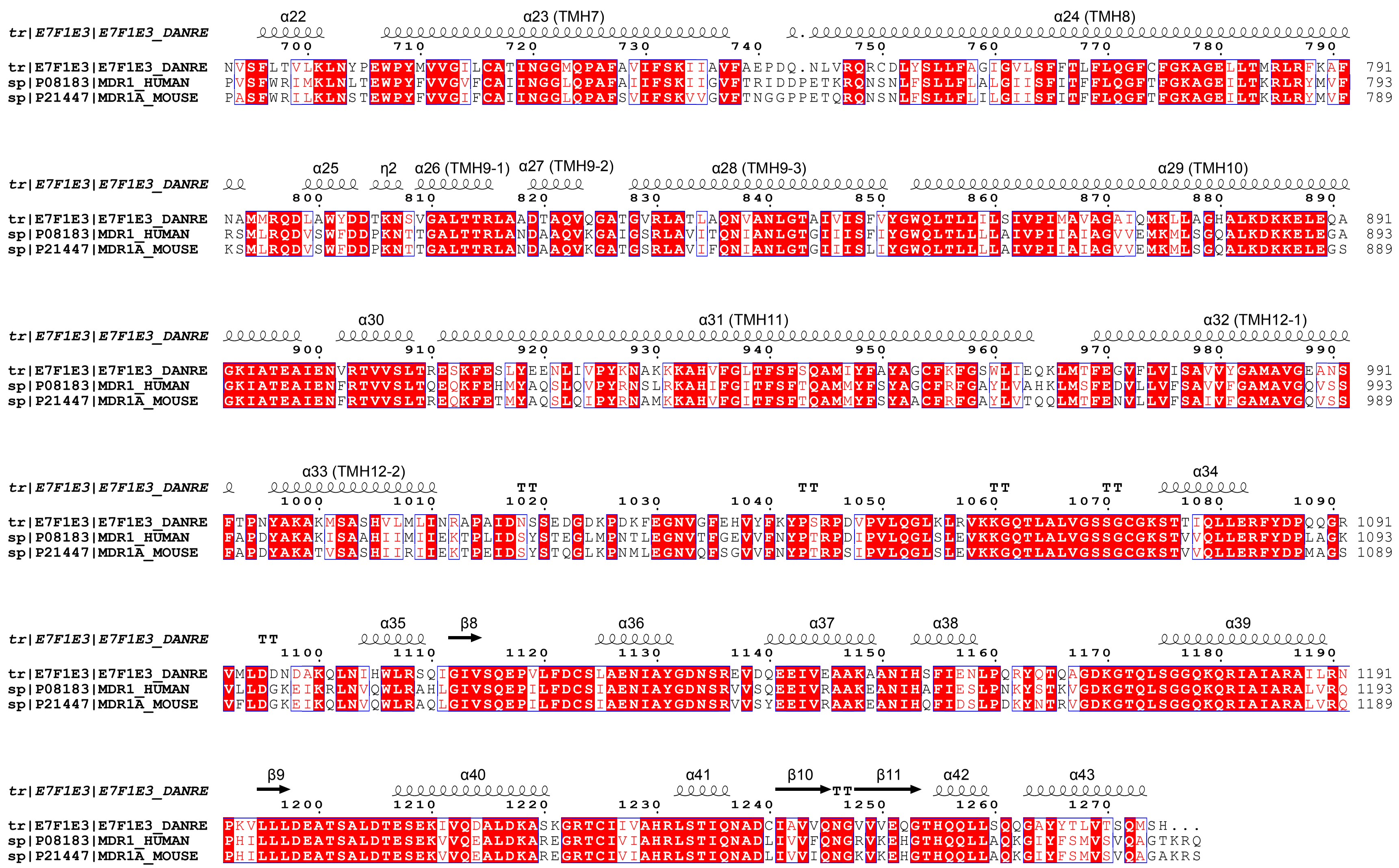

Zhan et al., Figure S4B

Figure S4. Sequence alignment of zebrafish Abcb4, hP-gp, and mP-gp. Secondary structure assignments based on Apo Abcb4 structure are placed above the alignment (A) N-terminal half. (B) C-terminal half.

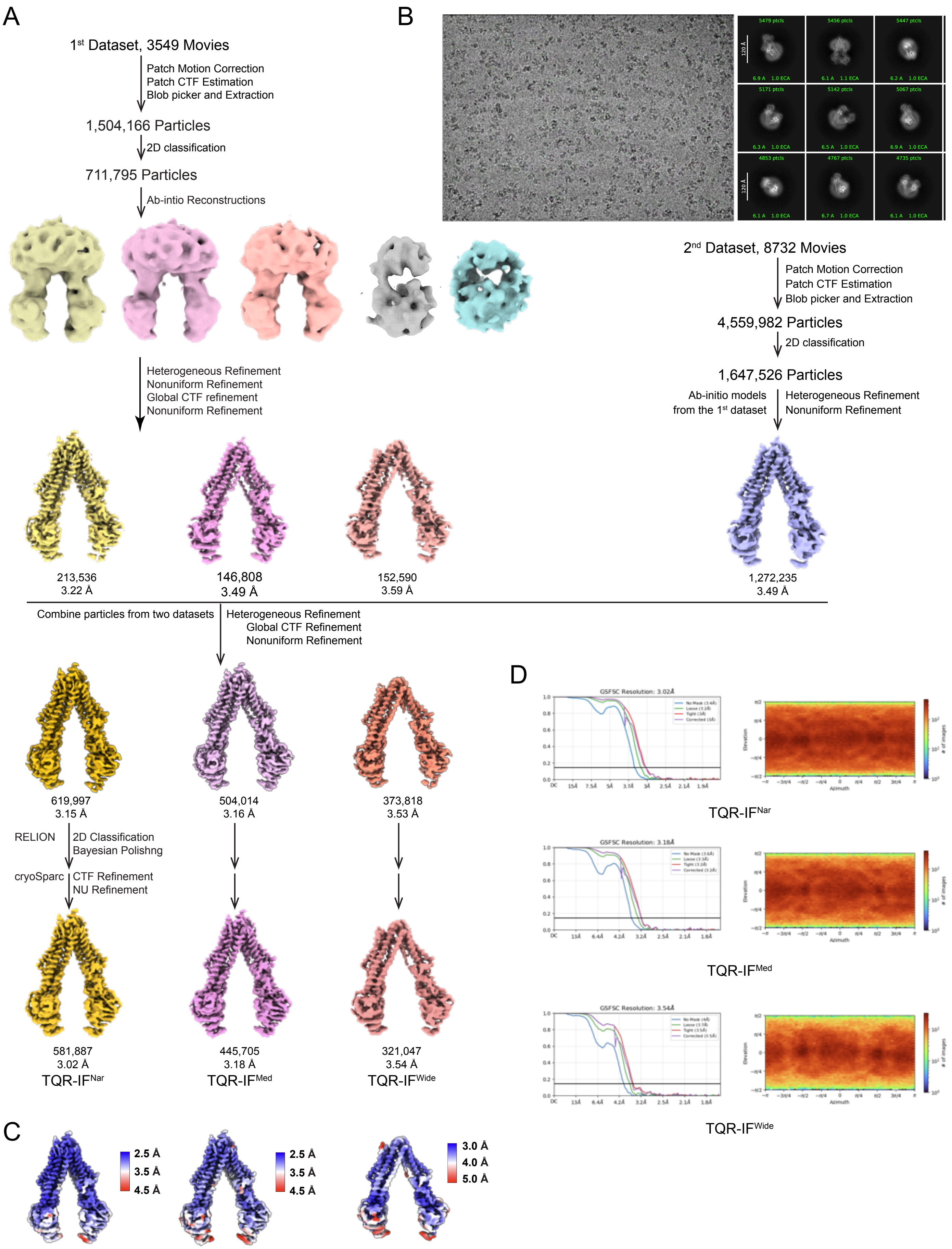

Zhan et al., Figure S5

Figure S5. Cryo-EM data processing workflow for DrAbcb4/tariquidar. The final dataset includes (A) subset 1 with 3,549 movies and (B) subset 2 with 8,732 movies, collected from the same DrAbcb4 sample incubated with tariquidar, ATPγS, and $MgCl_2$. Representative raw micrograph and 2D class averages are given for the 2nd data set. Particles from the two data sets were combined. 3D classifications and reconstructions obtained are shown. (C) Final 3D maps of the three major conformations, colored by local resolution. (D) Gold-standard FSC curves (left) and viewing direction distribution plots (right) for the three final reconstructions.

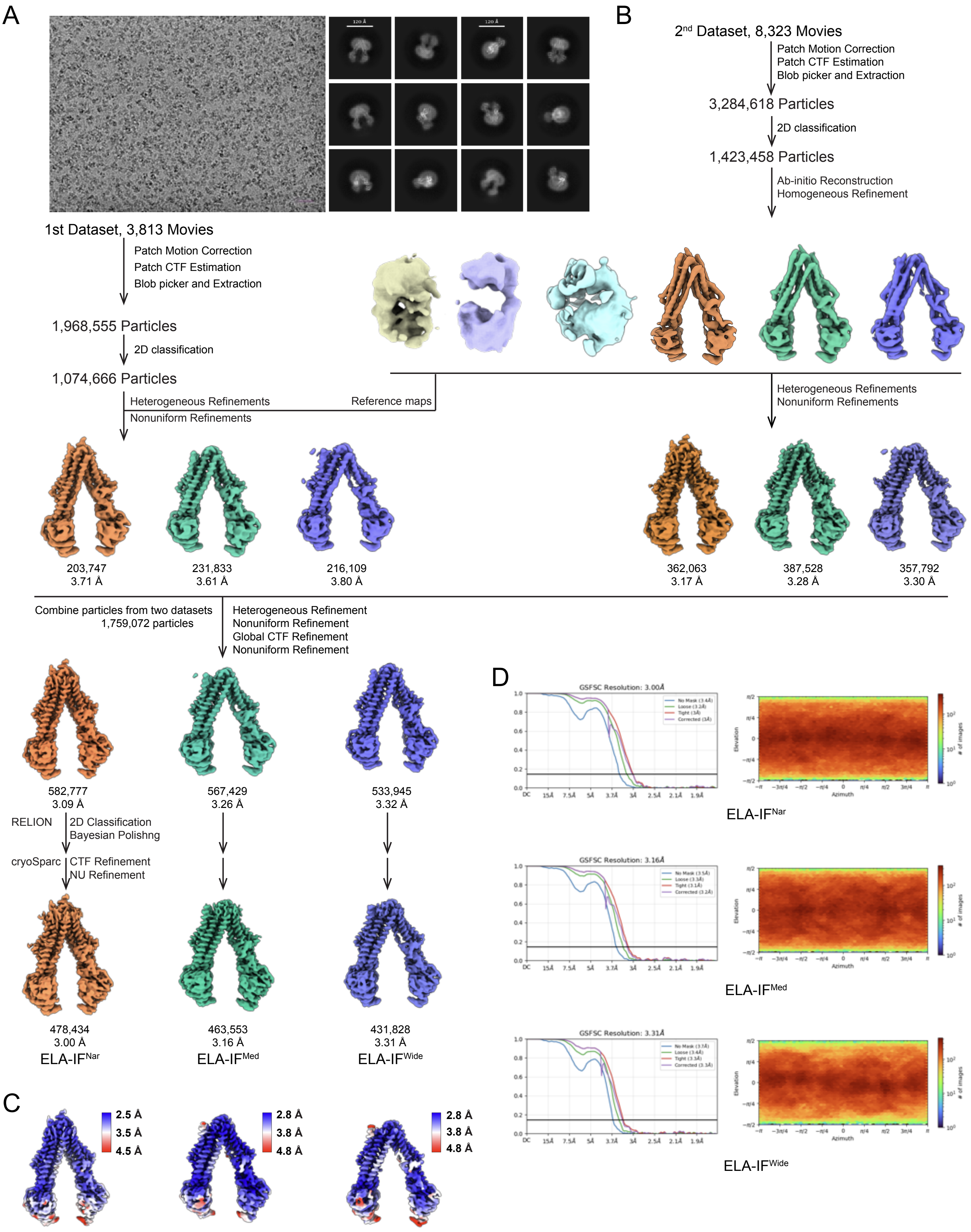

Zhan et al., Figure S6

Figure S6. Cryo-EM data processing workflow for DrAbcb4/elacridar dataset. The final dataset comprises (A) a smaller subset with 3,813 movies and (B) a larger subset with 8,323 movies, collected from the same DrAbcb4 sample incubated with elacridar, ATPγS, and $MgCl_2$. Representative micrograph and 2D class averages for the 1st data set are shown. Particles from the two data sets were combined. 3D reconstructions from the data processing workflow are shown. (C) Final 3D maps of the three major conformations, colored by local resolution. (D) Gold-standard FSC curves (left) and viewing direction distribution plots (right) for the three final reconstructions.

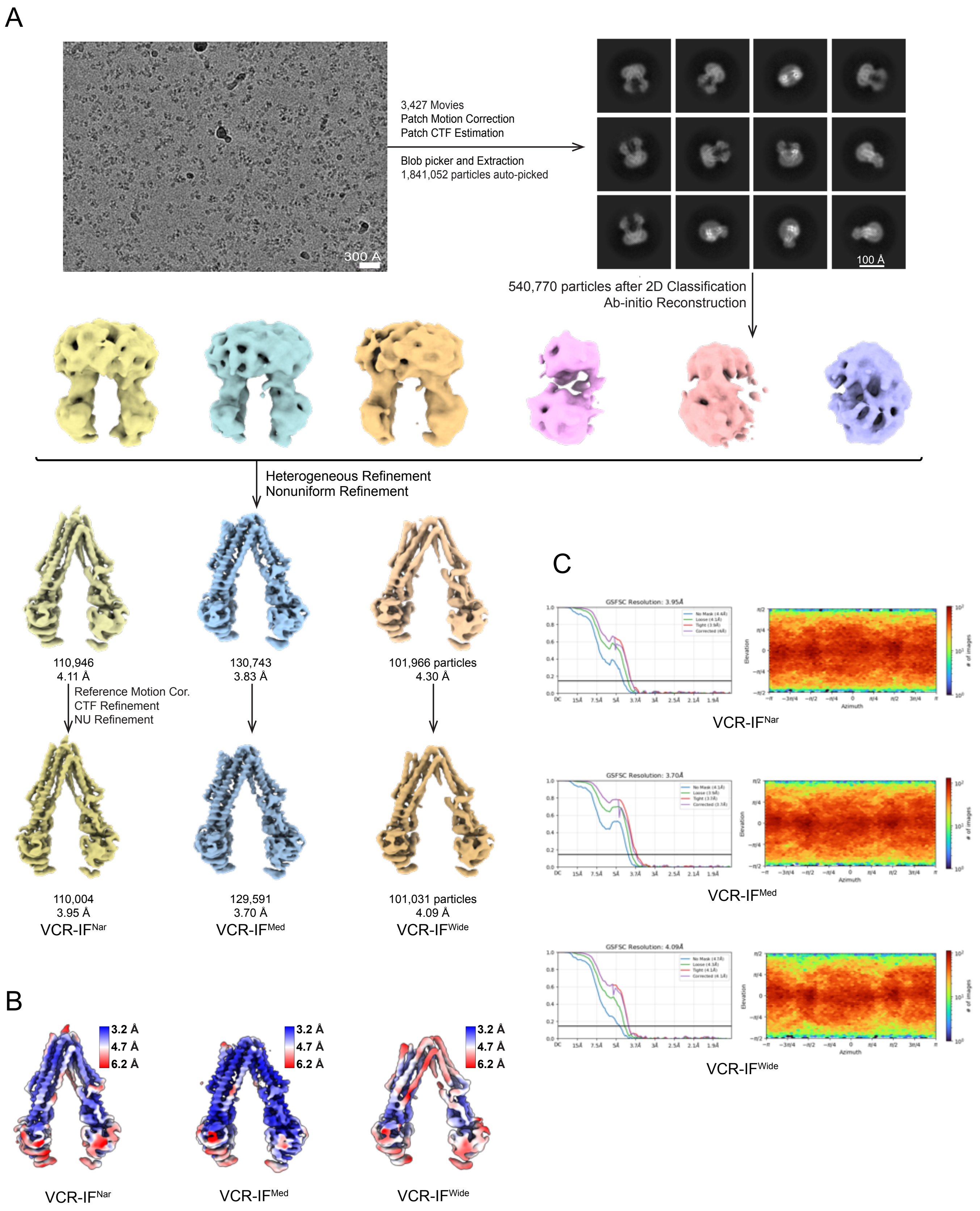

Zhan et al., Figure S7

Figure S7. Cryo-EM data processing workflow for DrAbcb4/vincristine dataset. (A) Representative micrograph, 2D class averages, and 3D reconstructions from the DrAbcb4/vincristine dataset are shown. Three major conformations with resolutions between 3.7 Å to 4.1 Å were obtained. (B) Final 3D reconstructions of the three conformations, colored by local resolution. (C) Gold-standard FSC curves (left) and viewing direction distribution plots (right) for the three final reconstructions.

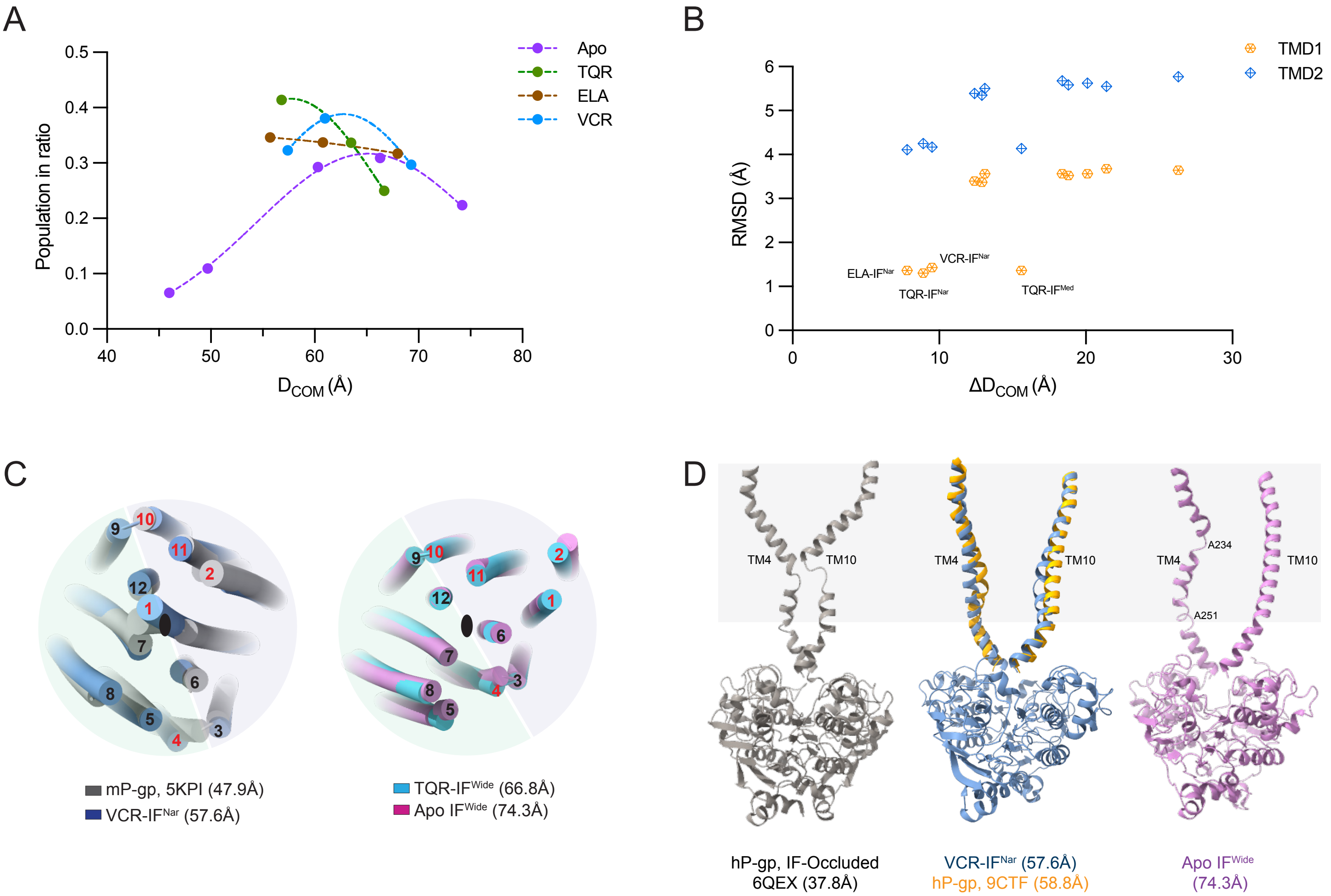

Zhan et al., Figure S8

Figure S8. Structural deviations of P-gp and DrAbcb4 as a function of D_{COM}. (A) DrAbcb4 conformational landscape depicted as the normalized particle numbers as a function of D_{COM} can be modulated by presence of substrate/inhibitor. (B) Using mP-gp (PDB:5KPD) as a reference for superposing the 12 DrAbcb4 structures pairwise, the rms deviations (RMSD) are plotted against the changes in D_{COM} (ΔD_{COM}), revealing a positive correlation indicative of coordinated movement. (C) Top view of the TMDs from the extracellular side, with the TMD1 shaded in light grey and TMD2 shaded in light green. Comparing structures with D_{COM} of 47.9 Å and 57.6 Å (Left) to those of 66.8 Å and 74.3 Å (Right), several TMHs shifted places indicating helix repacking. (D) Conformation of TMHs 4 and 10 at different D_{COM} values. For clarity, the rest TMHs are not shown.

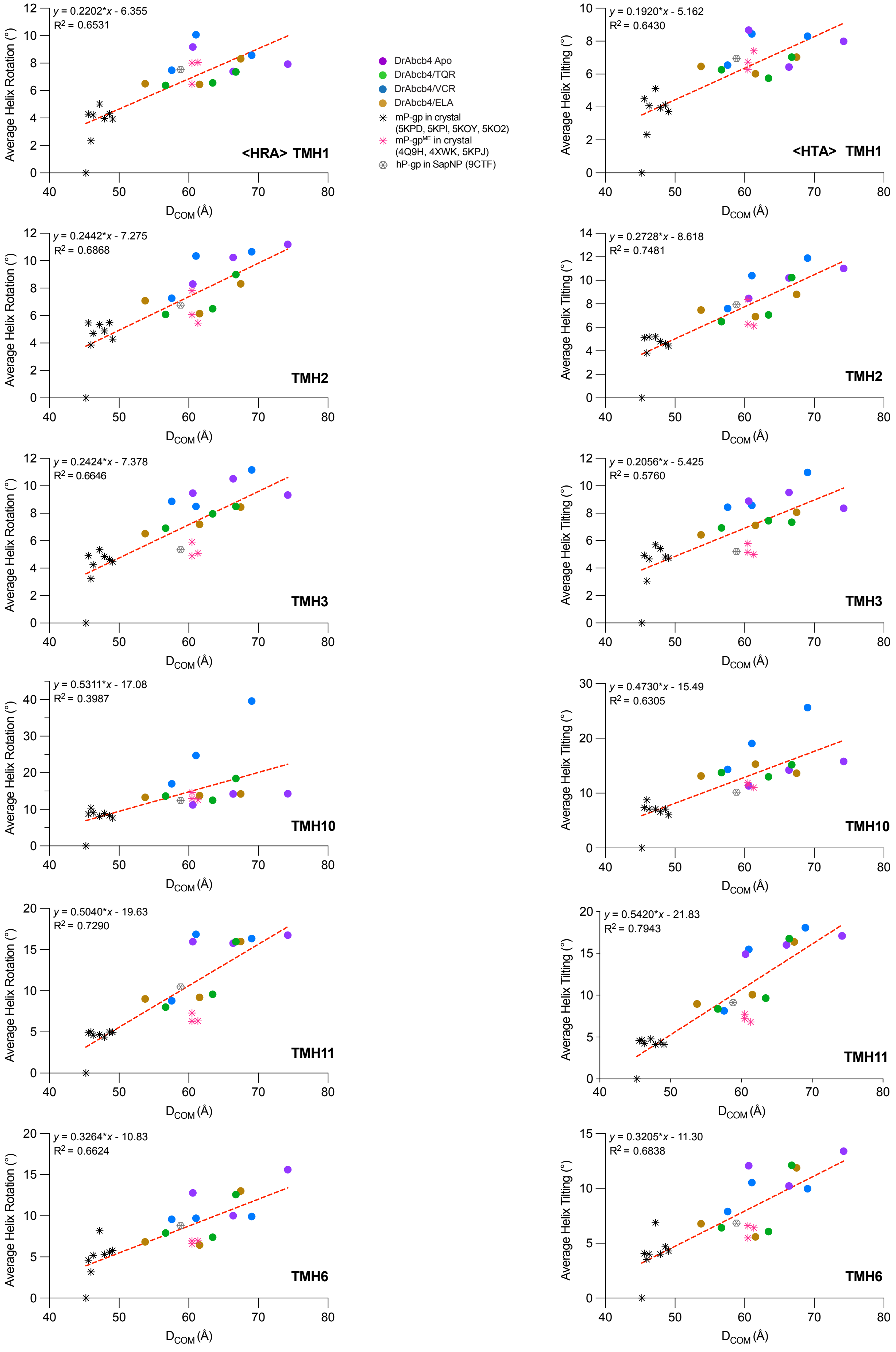

Zhan et al., Figure S9A

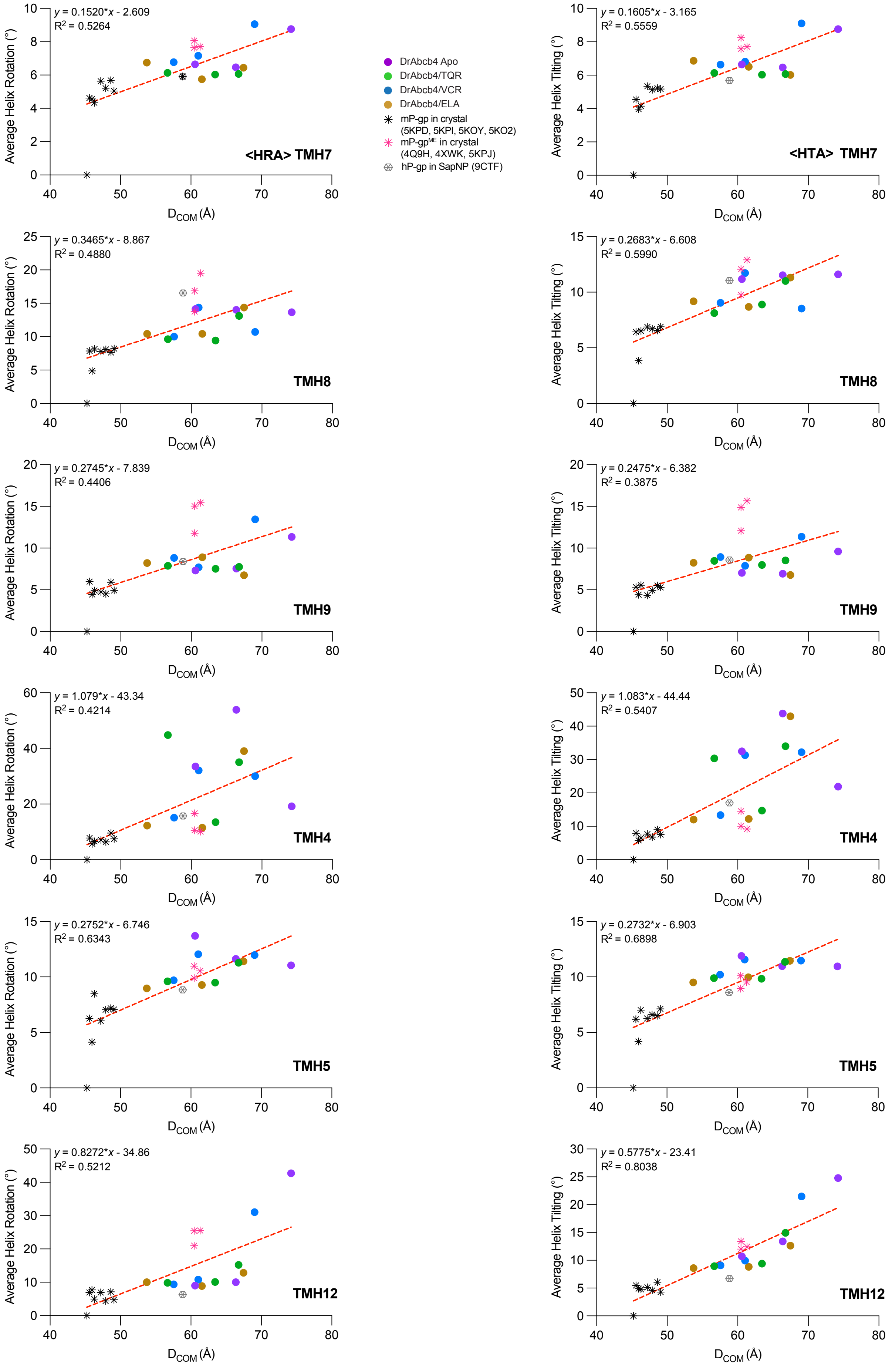

Zhan et al., Figure S9B

Figure S9. Scatter plots showing the correlation of D_{COM} with average rotation angle <HRA> and average tilting angle <HTA> of individual TM helices in TMD1 (Figure S9A) and in TMD2 (Figure S9B). Left: Scatter plot of <HRA> as a function of D_{COM}. Left: Scatter plot of <HTA> as a function of D_{COM}. The dashed lines are trending lines through the sample dots. Fitting equations and R values are also given.

	TMD1	hP-gp	mP-gp	DrAbcb4
	TM1	L65	L64	L70
	TM1	M68	M67	M73
	TM1	M69	M68	V74
	TM1	F72	F71	F77
	TM6	F336	F332	F341
	TM6	V338	V334	V343
	TM6	L339	L335	L344
	TM6	I340	I336	I345
	TM6	A342	A338	A347
	TM6	F343	F339	F348
	TM6	S344	S340	G349
*	TM6	G346	G342	G351
	TM6	Q347	Q343	Q352
	TM6	P350	P346	P355
	TM10	E875	E871	Q873
	TM10	M876	M872	M874
	TM10	L879	L875	L877
	TM10	S880	S876	A878
*	TM11	T945	T941	S943
	TM11	Q946	Q942	Q944
	TM11	M949	M945	I947
	TM11	Y950	Y946	Y948
	TM11	Y953	Y949	Y951

DrAbcb4	mP-gp	hP-gp	TMD2	
N720	N717	N721	TM7	
Q724	Q721	Q725	TM7	
F727	F724	F728	TM7	
A728	S725	A729	TM7	
F731	F728	F732	TM7	
F757	F755	F759	TM8	
G761	G759	G763	TM8	
S764	S762	S766	TM8	
F765	F763	F767	TM8	
L768	F766	F770	TM8	
Q771	Q769	Q773	TM8	
V829	S827	S831	TM9	
A832	A830	A834	TM9	
Q836	Q834	Q838	TM9	
I223	I214	I218	TM4	
V226	I217	I221	TM4	
S227	S218	S222	TM4	
P228	P219	P223	TM4	*
L230	L221	L225	TM4	
G231	G222	G226	TM4	*
A234	A225	A229	TM4	
A235	G226	A230	TM4	*
I237	W228	W232	TM4	
G238	A229	A233	TM4	
K239	K230	K234	TM4	*
M241	L232	L236	TM4	
T242	S233	S237	TM4	
F244	F235	F239	TM4	
V300	A291	A295	TM5	*
M304	M295	I299	TM5	
T307	A298	A302	TM5	*
F308	F299	F303	TM5	
I311	I302	I306	TM5	
Y312	Y303	Y307	TM5	
S314	S305	S309	TM5	
Y315	Y306	Y310	TM5	
I976	F974	F978	TM12	
S977	S975	S979	TM12	
Y981	F979	F983	TM12	
M984	M982	M986	TM12	
A985	A983	A987	TM12	
E988	Q986	Q990	TM12	
A989	V987	V991	TM12	
F992	F990	F994	TM12	

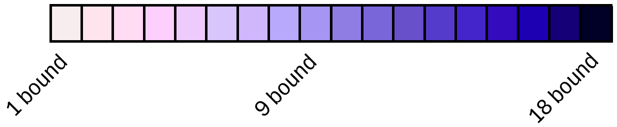

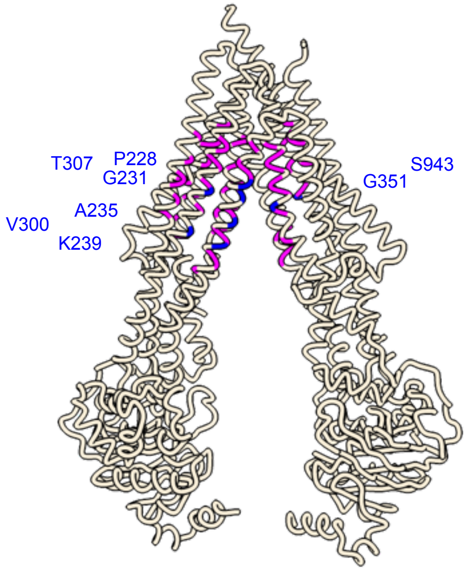

Zhan et al., Figure S10

Figure S10. Sequence conservation for residues interacting with bound compounds. The left panel lists all drug-interacting residues from TM helices of TMD1, and the right panel shows those from TM helices of TMD2. Corresponding residues in hP-gp, mP-gp, and DrAbcb4 sequences are given with colored squares indicating the frequency of each residue found interacting with bound compounds. Newly identified drug-interacting residues in DrAbcb4 structures are marked with an asterisk, and their locations are highlighted in blue in the ribbon diagram of DrAbcb4.

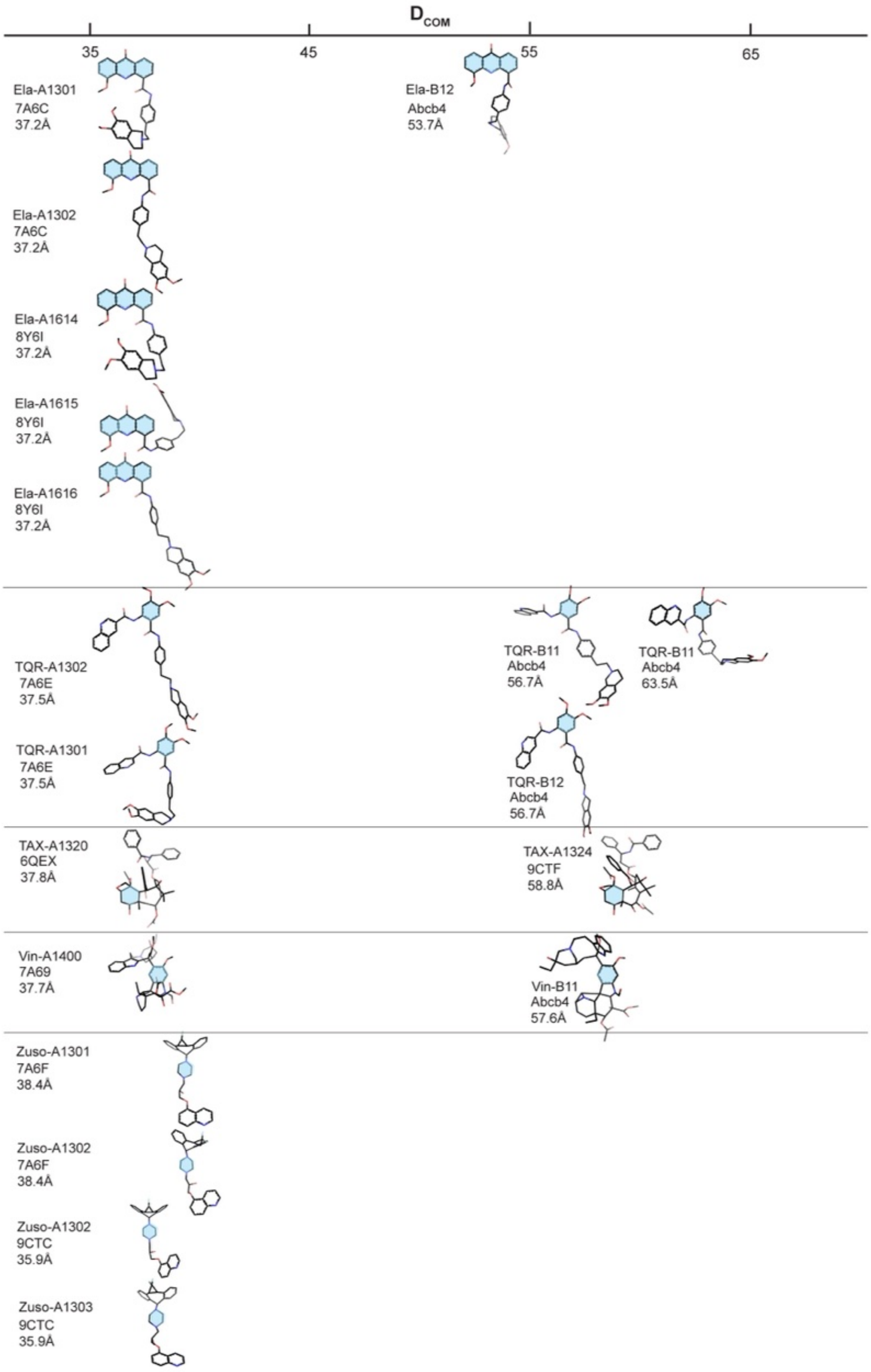

Zhan et al., Figure S11

Figure S11. Poses of bound compounds arranged by D_{COM} values. Compounds are illustrated as stick models and labelled with their PDB codes and D_{COM} values. The chemical moieties that were used for alignment are colored cyan.
