# Supplementary material for: Functional implications of the conformational landscape of a multidrug transporter revealed by Zebrafish Abcb4 structures
Source: Nat Commun. 2026 May 30;17:7014. doi: 10.1038/s41467-026-73751-4 (PMC13392234; doi:10.1038/s41467-026-73751-4)
Supplement: Supplementary file 2 — Description of Additional Supplementary Files [file 41467_2026_73751_MOESM2_ESM.pdf]

Description of Additional Supplementary Files

File name: Supplementary Movie 1
Description: 3D Variability Analysis for the DrAbcb4 apo dataset.

File name: Supplementary Movie 2
Description: 3D Variability Analysis for the DrAbcb4/Tariquidar dataset.
