# Supplementary material for: Functional implications of the conformational landscape of a multidrug transporter revealed by Zebrafish Abcb4 structures
Source: Nat Commun. 2026 May 30;17:7014. doi: 10.1038/s41467-026-73751-4 (PMC13392234; doi:10.1038/s41467-026-73751-4)
Supplement: Supplementary file 6 — Transparent Peer Review file [file 41467_2026_73751_MOESM6_ESM.pdf]

nature portfolio

Peer Review File

Functional Implications of the Conformational Landscape of a Multidrug Transporter Revealed by Zebrafish Abcb4 Structures

Corresponding Author: Dr Di Xia

This file contains all reviewer reports in order by version, followed by all author rebuttals in order by version.

Version 0:

Reviewer comments:

Reviewer #1

(Remarks to the Author)
ABCB4

Zhan and coworkers investigate the conformational spectrum of the zebrafish ABC transporter ABCB4 in response to several compounds. Zebrafish ABCB4 (DrABCB4) serves as a model system for the human ABC transporter ABCB1 or P-glycoprotein (hP-gp), as a functional homolog, exhibiting comparable basal and stimulated ATPase activities.
In total, the authors present 12 structures comprising apo, tariquidar-, elacridar-, and vincristine-bound inward-facing conformations.

The value of the study is, in my view, more about how DrABCB4 interacts with specific compounds than the translational value, which assumes that substrate interactions can be directly related between hP-gp and DrABCB4, especially in light of already different substrate interactions between human and mouse P-gp.

Using their new and already published structures, the authors analyse and compare the conformational spectrum and the underlying rotations and movements of individual helices and the NBDs. Ultimately, these changes modulate the shape of the substrate binding pocket, which could explain polyspecificity. Such analyses are instrumental to systematically understand the changes underlying the ABC transporters' mechanism. Given the large number of already published structures, including some very recent ones from cryo-EM, it is desirable that these also be incorporated in the analysis. Overall, the authors propose a model based on fluid binding sites that is capable of providing just the right environment for various drugs.

The manuscript contains useful information and high-quality structural data. However, many of the conclusions drawn are generic and not tailored or necessarily emerging from the provided data. For example, even without the additional structures provided in this work, the proposed fluidic model can be proposed, and the additional information provided by the new structures is comparatively small.
Despite the apo datasets, further appropriate controls, which I acknowledge may not in every case be easy to come by, validating the claims specifically is lacking. Furthermore, the authors need to more effectively utilize their data to make their claims. Additionally, the analysis of the conformations and comparison to available structures falls short and should be expanded, including many more of the available P-gp structures. Currently, the paper could be condensed to: We see NBD distance changes modulating the TMDs, which in turn reshape the binding pocket, which could explain polyspecificity. I think everybody can agree with this; this is neither novel, nor would we need the provided data to make this claim. I believe that there are many more insights in these structures, which should be used to support and expand the hypothesis.

Specific comments:
While the authors mention conformational changes in the helices at many points in the manuscript, they remain comparably unspecific. When I analyzed the structures provided with the review, I noticed that in some conformations, TM2 moves into an unusual position (for example, VCR Med and Wide compared to VCR narrow), and entirely rearranges the geometry of the dimer interface. The authors have to consider this remarkable effect in more detail and perform the required experiments

to validate whether this is a detergent-induced effect. I want to be clear that I do believe that these conformations are functionally and physiologically relevant; however, this has to be explored more carefully.

The authors state that the NBD dimerization without substrates is very unlikely and that substrate binding is required to achieve dimerization in amounts measurable by cryo-EM. Line 168: This suggests that nucleotide binding alone is incapable of inducing NBD-dimerization in wild-type DrAbcb4 in the absence of substrate, at least not to a degree detectable by cryo-EM under our conditions. By contrast, structures of mP-gp with ATP/Mg2+ (PDB:8AVY, L335C+E/Q mutations) in detergent solution21 and hP-gp with ATP S/Mg2+ (PDB:9CTG) embedded in nanodisc30, both in outward facing (OF) conformation, were obtained in the absence of substrate.
Here, the authors must provide additional experiments before this claim can be made. First of all, the authors used ATPgammaS, a non-hydrolizable analogue of ATP. It is not certain that this substitute can sufficiently dimerize the NBDs. Especially, as even when ATP is added (turnover), NBD dimerization is not detectable – even though substrate is present. Only recently, a study showed such an OF-conformation in cryo-EM. But also there, only minute OF fractions were detectable in cryo-EM under turnover conditions using ATP as nucleotide. The cited experiments (L170…) were made under different conditions than (mutant /nanodisc) and are thus not comparable.
Thus, the claim that ATP binding alone is insufficient to dimerize DrABCB4 efficiently is not supported. EQ-mutations, different environments, and the usage of alternative analogues such as ATPVi or AMP-PnP should be explored to validate the hypothesis. And make it comparable to the other experiments. After all, the protein has basal ATPase activity – where should this come from if not from NBD-dimerization?

The authors suggest that two of the tested drugs (elacridar and tariquidar) stabilize the IF conformation, whereas vincristine destabilizes the protein. They base this claim on the different resolutions that they were able to obtain in the cryo-EM reconstructions. This is an interesting finding, but it also demands a careful statistical analysis. In general, it is not easy to get statistics between different cryo-EM datasets as numerous factors such as ice thickness, particle concentration and orientation, CTF-values, and number of particles in the final densities, all together have a significant impact on the achievable resolution. In analogy to biochemical experiments, biological replicates would be required to validate such an interpretation. As cryo-EM data collection is expensive and time-consuming, the acquisition of biological replicates may not always be possible; therefore, it is essential to mine the dataset as much as possible to consolidate the findings. An acceptable option could be the generation of datasets into comparable groups across the obtained datasets. Through this, at least CTF-values and particle numbers, etc., can be homogenized, which would provide a more reliable statistic.

The authors emphasize that their conformations are unrestricted by means of Antibodies, X-tal packing, and nanodiscs. While I generally agree with this statement, it remains to be shown that the conformations are also not restricted by the detergent (DDM) used in their study. For MsbA, it has recently been shown that the used hydrophobic environment influences the degree of NBD separation – I am not sure how this translates to other ABC transporters, but it is worthwhile to consider that such effects may also happen for DrABCB4. Thus, the above statement may not be 100% true and needs to be validated.

The authors describe conformational adaptations and suggest that rather than the transport substrate binding affecting the NBD distance, nucleotide binding affects the NBD distance. The authors conclude that the various conformations serve as a versatile binding site, explaining poly specificity. If this were true, the authors need to show that all these conformations exist in the apo state. This would certainly deserve a separate figure.

In general, the authors need to be more specific when describing the conformations. Their data is rich and insufficient to address conformational changes globally, but they must investigate the underlying shift in detail.

Minor Comments

It would serve readability if the authors were more specific in some sections of the manuscript. For example, in line 264 “We noticed that in drug-bound structures
such as DrAcb4/TQR, some helices display large <HRA> and <HTA> values, deviating
significantly from trending lines, indicating involvement of residues in these helices in drug binding.” I would immediately like to know which helices are being discussed, and I do not think that this is adding to clarity.

I do not think that the authors show this:
Line 380: In this study, the similarity extends beyond function and structure to include the mechanism of function, as revealed by the structures of DrAbcb4, both drug-free and drug-bound, in the presence of ATPS/Mg2+

Line 43: ATP hydrolysis by the NBDs drives conformational changes of the TMDs, enabling substrate binding and release on opposite sides of the membrane. This statement is incorrect, as it has been shown several times that already ATP binding drives the conformational change.

Figure1 and 2 – should be improved for clarity. The structures and models are tiny, and so is the text. Neither is easy to understand.

Reviewer #2

(Remarks to the Author)
an anonymised report, please.

comments:
This is a nice manuscript revealing unconstrained cryo-EM structures of multiple zebrafish abcb4 (unlike the many constrained structures of human ABCB1), an ortholog of human ABCB 1that appears to be for the most part functionally similar to human ABCB1 in that abcb4 is localized to the zebrafish blood-brain barrier. With respect to orthologs, there are differences among rodent, human and zebrafish P-gps in that their linkers are of different length. This seems important because length of the linker affects both basal and substrate stimulated activity. In this respect it would be nice if the authors could address if this (or other factors) account for differences between abcb4 and abcb1 with respect to verapamil stimulation and inhibition by cyclosporin. Do differences in linker account for this or are there other factors? Human Abcb1 when comparing the apo to substrate-bound structure show large, asymmetric rearrangements within the membrane. Some of these changes are thought to have a gating function, perhaps through effects on an autoinhibitory TM? It would be helpful to know if similar changes and analogous domains are operative in abcb4. If not, please account for why not. Additionally, some determinations of how the size and volume of the binding pocket change would be important to include and comment if this is related to packing of the TMD. Elacridar shows inhibition whereas tariquidar appears not to. While the authors propose helix rotation and tilt as important features associated with ligand binding it is hard to reconcile these disparate effects. Are there dramatic differences in interaction with key amino acid residues for these two inhibitors? Does tariquidar actually behave more like a substrate for Abcb4 and are there marked differences in the contact frequency of key residues? It appears that elacridar affect on the NBD separation is more similar to vincristine than to tariquidar. Thoughts on this?

Version 1:

Reviewer comments:

Reviewer #1

(Remarks to the Author)
The authors made a solid effort and addressed almost all of my comments. However, aside from an attempt to reconstitute DrAbcb4 into a nanodisc, the authors largely refrained from performing the required additional experiments. For many of my comments, this is acceptable, but I am surprised that my comment 7 is not properly addressed. I think I opened an avenue to experimentally validate this claim, even suggesting a simple processing-based experiment that could have been performed without collecting additional data or generating new samples.
The literature is full of claims where lower achievable resolution in a single cryo-EM experiment is equated with greater flexibility. This can obviously be true, but often wrong. It has to be validated! The vinvristine dataset contains a third of the raw micrographs and a third of the particles in each map. Thus, lower resolution is to be expected - regardless of flexibility.
I suggested a facile experiment that could directly address this claim and validate it (or not). I am puzzled by the authors' preference to evade this obvious solution to their experimental data and instead rephrase the text.

Reviewer #2

(Remarks to the Author)
The authors have improved the manuscript. I have no additional amendments.

Version 2:

Reviewer comments:

Reviewer #1

(Remarks to the Author)
The authors now did the suggested control, and it turns out that their original claim was not substantial. They reverted this in the manuscript, and that's okay with me. I am a bit surprised about their wording, though, as they should be thankful to me for figuring this out. Too many papers in structural biology make claims without appropriate controls. It is very important that we, as a field, implement such necessary steps. Frankly, I am quite unhappy that it took two rounds of reviews before the authors performed this required, but simple, experiment. Once a claim is in the literature, it is very difficult to present something opposing, even if it is correct.

Open Access This Peer Review File is licensed under a Creative Commons Attribution 4.0 International License, which permits use, sharing, adaptation, distribution and reproduction in any medium or format, as long as you give appropriate credit to the original author(s) and the source, provide a link to the Creative Commons license, and indicate if changes were made.
In cases where reviewers are anonymous, credit should be given to 'Anonymous Referee' and the source.
The images or other third party material in this Peer Review File are included in the article's Creative Commons license, unless indicated otherwise in a credit line to the material. If material is not included in the article's Creative Commons license and your intended use is not permitted by statutory regulation or exceeds the permitted use, you will need to obtain permission directly from the copyright holder.
To view a copy of this license, visit https://creativecommons.org/licenses/by/4.0/

Nature Communications

REVIEWER COMMENTS

Reviewer #1 (Remarks to the Author):

ABCB4

Comment (1): Zhan and coworkers investigate the conformational spectrum of the zebrafish ABC transporter ABCB4 in response to several compounds. Zebrafish ABCB4 (DrABCB4) serves as a model system for the human ABC transporter ABCB1 or P-glycoprotein (hP-gp), as a functional homolog, exhibiting comparable basal and stimulated ATPase activities.
In total, the authors present 12 structures comprising apo, tariquidar-, elacridar-, and vincristine-bound inward-facing conformations.

The value of the study is, in my view, more about how DrABCB4 interacts with specific compounds than the translational value, which assumes that substrate interactions can be directly related between hP-gp and DrABCB4, especially in light of already different substrate interactions between human and mouse P-gp.

Response: Prior work [1, 2] has demonstrated that DrAbcb4 is similar to human ABCB1 in tissue distribution, amino acid sequence especially of the NBDs, ATPase activity, and strikingly similar in substrate profile. For this work, our focus is on demonstrating structural and mechanistic similarities between DrAbcb4 and human ABCB1, illustrating the value of DrAbcb4 as a model system for studying the mechanism of P-gp function.

In terms of translational value, it is well recognized that many drugs and treatments in the clinic and in clinical trials can trace their origin to zebrafish [3]. This is because of the well-established advantages for using zebrafish as a vertebrate model organism including similar tissue development, 70% similarity of genomes, 80% shared diseases, availability of various disease models, rapid growth, etc. It has been shown that DrAbcb4, mouse, and human ABCB1 share common inhibitor and substrate profiles. For example, human and mouse P-gp inhibitors such as tariquidar, valspodar, elacridar, and verapamil were shown to inhibit DrAbcb4 effectively [1]. Furthermore, DrAbcb4 KO animals exhibit very similar phenotypes to their mouse counterpart [2]. Thus, it is highly likely that an inhibitor identified using the DrAbcb4 screening system would also inhibit mouse and human ABCB1. The recently published EM structure of yellowfin tuna Abcb4 in complex with pesticide DDT [4] confirmed previous observation that DDT inhibited human P-gp transport activity [5]. In the present work, we also show that amino acid residues involved in contacts with substrates and inhibitors have higher sequence conservation than the overall sequence (Figure S10). More importantly, the proposed dynamic sampling model or fluid hypothesis of substrate binding may be a common feature to MDR transporters, in which substrate binding relies less on specific residue interactions but more on combined electrochemical and geometric property of the binding site. Therefore, it seems reasonable to assume the potential translational value of zebrafish Abcb4 in searching for inhibitors of P-gp, even if the specific interactions of the same inhibitor with DrAbcb4 and P-gp may differ.

Comment (2): Using their new and already published structures, the authors analyse and compare the conformational spectrum and the underlying rotations and movements of individual helices and the NBDs. Ultimately, these changes modulate the shape of the substrate binding pocket, which could explain polyspecificity. Such analyses are instrumental to systematically understand the changes underlying the ABC transporters' mechanism. Given the large number of already published structures, including some very recent ones from cryo-EM, it is desirable that these also be incorporated in the analysis. Overall, the authors propose a model based on fluid binding sites that is capable of providing just the right environment for various drugs.

Response: We appreciate the reviewer's positive comment on the relationship of conformational spectrum (open-and-close) of P-gp and dynamic nature of the substrate-binding surface, which is one of the main conclusions of our work. We also agree with the reviewer that more existing structures should be included in structural analyses. Although it is not our intention to claim that the fluid hypothesis a universal mechanism for all ABC transporters, this mechanism appears to be applicable to MDR ABCB sub-family ABC transporters.

In the submitted version, we did indeed include some published structures as representatives to cover the IF conformational spectrum of P-gp. For example, in Figure 3E, we included six published structures of P-gp in the OF conformation: 6C0V of hP-gp, and 7ZK4, 7ZK5, 7ZK6, 7ZKA and 8AVY of mP-gp. Similarly, eight structures of P-gp in the IF-occluded conformation were included in the analysis: 6QEX, 7A65, 7A69, 7A6C, 7A6E and 7A6F of hP-gp, and 6FN1 and 6QEE of chimeric human/mouse-P-gp. For IF conformations, four crystal structures of unmethylated mP-gp (5KPD, 5KPI, 5KOY and 5KO2, each has two chains in the asymmetric unit) and three methylated mP-gp (4Q9H, 4XWK and 5KPJ) were included in the analysis. A total of 33 PDBs with 37 unique P-gp structures were used in Figure 3E, which included 12 structures of DrAbcb4 from this work, 9 published crystal structures from 5 PDBs from our lab, and 16 published structures from other groups.

In the revision, we included more relevant structures from a recent publication [6], as suggested by the reviewer. In the revised Figure 3E, we further incorporated the following structures in the analysis: 9CTG (hP-gp in the OF conformation), 9CTC (hP-gp in the IF-occluded conformation), 9CR8 (hP-gp in the IF-Closed conformation), and 9CTF (hP-gp in the IF conformation). Also in the revision, we included 9CTF (hP-gp in the IF conformation, bound with Taxol) in the correlative analysis of IF conformations, leading to the revised Figure 3D, Table 1 and Supplementary Figure 9.

As our analysis was critically dependent on accurately positioned main chain atoms, we purposely only chose structures with relatively high resolution (better than 4 Å) and good model completeness, omitting models that have no NBDs, no side-chain atoms, or incomplete NBDs. We also added the PDB codes of these structures in the figure legend of 3D and 3E for clarity. In the text, we revised the sentence on page 11 from "To cover a larger D_{COM} range, we also included in this plot crystal structures of mP-gp and methylated mP-gp (mP-gp^{ME})" to " To cover a larger D_{COM} range, we also included crystal structures of mP-gp, methylated mP-gp (mP-gp^{ME}), and a few EM structures of mP-gp and hP-gp".

Comment (3): The manuscript contains useful information and high-quality structural data. However, many of the conclusions drawn are generic and not tailored or necessarily emerging from the provided data. For example, even without the additional structures provided in this work, the proposed fluidic model can be proposed, and the additional information provided by the new structures is comparatively small.

Response: We thank the reviewer for his/her appreciation of our "high-quality structural data", which we strive to achieve. We have a different view on the comment that "even without the additional structures provided in this work, the proposed fluidic model can be proposed". Without question, previous structural data have demonstrated the incredible dynamics of the substrate binding surface of P-gp. However, the majority of these data appeared in singularly clustered conformations. The novel insights drawn from the DrAbcb4 structures presented in this work are the following: (1) The observed dynamics of the substrate-binding surface is coupled to the open-and-close movement of the two lobes; Surface change is not just due to side chain movements. (2) TMDs are no longer conventional rigid bodies having fixed hydrophobic cores, as having been depicted in the literature. Rather, each individual helix undergoes dynamics independently. Prior studies often focused on the kinks of TM4 and TM10 and their likely role in gating, while in this work we described dynamics observed in all 12 TM helices. (3) The wide-open IF conformations with much extended separations of the two halves corroborate prior FRET and DEER measurements. (4) A more complete conformational landscape composed of DrAbcb4 structures in different IF states in one experiment in contrast to existing structures that are often clustered as for example in crystal structures. (5) Our structural data with bound tariquidar, elacridar, and vincristine showing these compounds in different binding poses and interacting with different binding environments from those of published work are indispensable for the formulation of the fluid hypothesis. (6) Substrate polyspecificity can now be more precisely defined at molecular level. In additional to being able to bind different, unrelated compounds, P-gp can also bind multiple compounds simultaneously in different protein conformations and bind compounds in different poses.

Comment (4): Despite the apo datasets, further appropriate controls, which I acknowledge may not in every case be easy to come by, validating the claims specifically is lacking. Furthermore, the authors need to more effectively utilize their data to make their claims. Additionally, the analysis of the conformations and comparison to available structures falls short and should be expanded, including many more of the available P-gp structures. Currently, the paper could be condensed to: We see NBD distance changes modulating the TMDs, which in turn reshape the binding pocket, which could explain polyspecificity. I think everybody can agree with this; this is neither novel, nor would we need the provided data to make this claim. I believe that there are many more insights in these structures, which should be used to support and expand the hypothesis.

Response: The authors agree with the reviewer that our analysis can include more published structures, which we added in the revision for our analyses (see the Answer to Reviewer's Comment (2) above). Following this suggestion, we expanded the analysis and comparison to available structures in the PDB, as shown in the revised Supplementary Figure 8. We also revised the text on page 10 as follows: “The movement of TMHs 4 and 10 of DrAbcb4 drives the SBP wide open, in stark contrast to the enclosed SBP induced by kinking of TMHs 4 and 10 observed in P-gp’s IF-Occluded state (Supplementary Figure 8D). The latter led to the proposed substrate gating mechanism[18].”

With respect to novelty, please refer to our response to Comment (3) above.

We also concur with the reviewer that "there are many more insights in these structures, which should be used to support and expand the hypothesis". Reflecting on an old wisdom in the structural biology community that new structures are like an unexplored mine full of surprises. Nevertheless, space is limited in an article, forcing us to focus on the main observations.

Specific comments:

Comment (5): While the authors mention conformational changes in the helices at many points in the manuscript, they remain comparably unspecific. When I analyzed the structures provided with the review, I noticed that in some conformations, TM2 moves into an unusual position (for example, VCR Med and Wide compared to VCR narrow), and entirely rearranges the geometry of the dimer interface. The authors have to consider this remarkable effect in more detail and perform the required experiments to validate whether this is a detergent-induced effect. I want to be clear that I do believe that these conformations are functionally and physiologically relevant; however, this has to be explored more carefully.

Response: We thank the reviewer for carefully inspecting our structures and pointing out the movement of TMH2 in the structures of DrAbcb4 with bound vincristine. In our submission, we depicted several TMHs in Fig. 2C, showing their changes as a function of the open-and-close movement. In particular, it also mentioned the changes of TMH1 and TMH2 on Page 10 as "Notably, as the two lobes open wider and D_{COM} increases, (1) TMH1 and TMH2 unwind at their tips on the extracellular side of the membrane, extending the unstructured ECL1 loop (Figs. 2C and 3A, Supplementary movie 2)."

In the revision, we added the following sentence to describe the unusual positional shift of TMH2 as the reviewer pointed out: "the TMH1 and TMH2 also moved further away from the central pseudo-2-fold symmetry axis particularly at the extracellular side, which invoked distinct asymmetry between the two TMDs. (Supplementary Figure 8C)"

To describe the conformational changes in the helices more specifically, we also added the following in boldface to the text on page 10: "(2) Both coupling helices TMH10 and TMH11 of TMD1 undergo large positional shifts, **with their extracellular portion repositioned closer to the central axis** (Fig. 3B and Supplementary Figure 8C). (3) By contrast, only TMH4 of the coupling helix pair TMHs 4/5 of TMD2 undergoes similar positional changes **towards the central axis** (Fig. 3C and Supplementary Figure 8C). **The movement of TMHs 4 and 10 of DrAbcb4 drives the SBP wide open, in stark contrast to the enclosed SBP induced by kinking of TMHs 4 and 10 observed in P-gp's IF-Occluded state (Supplementary Figure 8D). The latter led to the proposed substrate gating mechanism[18].**

Regarding the unusual positional shift of TMH2, it is worth pointing out that this positional shift takes place when the two lobes open wide and is not vincristine-binding specific, as it is also observed in apo DrAbcb4 structures. As for whether this is a detergent-induced effect and its physiological relevance, we agree with the reviewer that appropriate control experiments would be necessary to draw conclusions.

Perhaps, the best control experiment would be to obtain DrAbcb4 structures in their native lipid environment or *in vivo* on intact cell membrane. High-resolution structure determination of membrane proteins directly from intact cells is a technique currently under development. After this technique is more matured, we will apply it to P-gp and other membrane proteins in their true biological environment. Nonetheless, prior AFM experiments, under planar lipid conditions and not in nanodisc, reported the existence of such wide-open IF conformations [7, 8], which is consistent with our structural data and indicative of their functional/physiological relevance. Supporting this line of argument, observations on the wide-open conformation have been reported for the bacterial multidrug transporter MsbA in native membranes [9].

So far, published structures of P-gp obtained in either detergents or membrane environment didn't show major differences. For example, in a 2024 paper by Hamaguchi-Suzuki et al. [10], cryo-EM structures of P-gp bound to triple elacridar molecules were obtained in both detergent micelles (PDB:8Y6H) and in nanodiscs (PDB:8Y6I), and they are "almost identical". In their 2019 paper [11], Alam et al. also reported that for the zosuquidar-bound structure of nanodisc-embeded P-gp (PDB:6QEE), "the orientation and binding interactions of the zosuquidar molecules are similar to those previously observed in the disulfide-trapped, detergent-solubilized $ABCB1_{HM}$ structure". Furthermore, P-gp structures in OF conformation obtained in different detergent micelles (PDB: 6C0V, 7ZK4) were also similar to that obtained in Saposin lipid nanoparticles (PDB:9CTG).

Taking together, although we have taken in consideration that the novel observations obtained from DrAbcb4 structures may be a detergent-induced effect, there are sufficient experimental observations to indicate that the use of detergent in general does not interfere with the drug binding mode or a particular protein conformation of P-gp, and the wide-open IF conformation is physiologically relevant. Our continuing efforts to obtain structures of P-gp or its homologs in similar wide-open IF conformations in lipid conditions would ideally provide a firm answer to this question.

Comment (6): The authors state that the NBD dimerization without substrates is very unlikely and that substrate binding is required to achieve dimerization in amounts measurable by cryo-EM.

Line 168: This suggests that nucleotide binding alone is incapable of inducing NBD-dimerization in wild-type DrAbcb4 in the absence of substrate, at least not to a degree detectable by cryo-EM under our conditions. By contrast, structures of mP-gp with ATP/Mg2+ (PDB:8AVY, L335C+E/Q mutations) in detergent solution[21] and hP-gp with ATPγS/Mg2+ (PDB:9CTG) embedded in nanodisc[30], both in outward facing (OF) conformation, were obtained in the absence of substrate.

Here, the authors must provide additional experiments before this claim can be made. First of all, the authors used ATPgammaS, a non-hydrolizable analogue of ATP. It is not certain that this substitute can sufficiently dimerize the NBDs. Especially, as even when ATP is added (turnover), NBD dimerization is not detectable – even though substrate is present. Only recently, a study showed such an OF-conformation in cryo-EM. But also there, only minute OF fractions were detectable in cryo-EM under turnover conditions using ATP as nucleotide. The cited experiments (L170...) were made under different conditions than (mutant /nanodisc) and are thus not comparable.

Thus, the claim that ATP binding alone is insufficient to dimerize DrABCB4 efficiently is not supported. EQ-mutations, different environments, and the usage of alternative analogues such as ATPVi or AMP-PnP should be explored to validate the hypothesis. And make it comparable to the other experiments. After all, the protein has basal ATPase activity – where should this come from if not from NBD-dimerization?

Response: In the submitted version, we did not intend to make the claim that ATP binding alone is insufficient to dimerize DrAbcb4 NBDs, as we listed a published example showing this conformation has been obtained for the wild type hP-gp reconstituted into saposin nanodisc in the presence of ATPγS (PDB:9CTG). We further stated that "the spontaneous open-and-close motion of DrAbcb4 may represent an important feature of the transporter sampling for potential ligand at resting state, which may also form the basis for the basal ATPase activity"

The statement in the submitted text "This suggests that nucleotide binding alone is incapable of inducing NBD-dimerization in wild-type DrAbcb4 in the absence of substrate, at least not to a degree detectable by cryo-EM under our conditions" overinterprets our experimental observations. What we intended to say was that in the presence of nucleotide alone, DrAbcb4 particles are heavily populated in the IF open state as indicated by the reconstructions of our cryo-EM data, while the OF conformation is at most short-lived with a minor particle population that was not captured by our cryo-EM analysis. Indeed, this observation has been confirmed by distance distributions between the two NBDs investigated by various techniques such as FRET [12]. Nevertheless, we are aware that one P-gp structure in the OF conformation has been obtained in the presence of nucleotide alone in the literature (PDB:9CTG).

In the revision on page 7, we replaced it with "This indicated that nucleotide binding alone did not yield a sufficient number of particles in the NBD-dimerized state to enable classifications and 3D reconstruction under our conditions".

Comment (7): The authors suggest that two of the tested drugs (elacridar and tariquidar) stabilize the IF conformation, whereas vincristine destabilizes the protein. They base this claim on the different resolutions that they were able to obtain in the cryo-EM reconstructions. This is an interesting finding, but it also demands a careful statistical analysis. In general, it is not easy to get statistics between different cryo-EM datasets as numerous factors such as ice thickness, particle concentration and orientation, CTF-values, and number of particles in the final densities, all together have a significant impact on the achievable resolution. In analogy to biochemical experiments, biological replicates would be required to validate such an interpretation. As cryo-EM data collection is expensive and time-consuming, the acquisition of biological replicates may not always be possible; therefore, it is essential to mine the dataset as much as possible to consolidate the findings. An acceptable option could be the generation of datasets into comparable groups across the obtained datasets. Through this, at least CTF-values and particle numbers, etc., can be homogenized, which would provide a more reliable statistic.

Response: We agree with the reviewer that the resolution limit of a reconstruction is determined by many factors as listed by the reviewer, of which protein conformation stability is one of them.

In our submitted text, we stated " Interestingly, reconstructions of the DrAbcb4/TQR and DrAbcb4/ELA datasets reached higher resolutions than the drug-free DrAbcb4, while those from the DrAbcb4/VCR

dataset had relatively lower resolutions (Supplementary Table 1), which suggests a potential stabilizing role of inhibitors, but not substrates, to the conformations of DrAbcb4 in solution"

In the revision, this sentence was revised to "Reconstructions from the DrAbcb4/TQR and DrAbcb4/ELA datasets reached higher resolutions than the drug-free DrAbcb4, while those from the DrAbcb4/VCR dataset had relatively lower resolutions (Supplementary Table 1)."

Comment (8): The authors emphasize that their conformations are unrestricted by means of Antibodies, X-tal packing, and nanodiscs. While I generally agree with this statement, it remains to be shown that the conformations are also not restricted by the detergent (DDM) used in their study. For MsbA, it has recently been shown that the used hydrophobic environment influences the degree of NBD separation – I am not sure how this translates to other ABC transporters, but it is worthwhile to consider that such effects may also happen for DrABCB4. Thus, the above statement may not be 100% true and needs to be validated.

Response: We think that the reviewer here refers to the recently published beautiful work on MsbA conformational spectrum by cryo-EM [13], in which detergents like DDM were shown to limit MsbA in the IF-wide conformation, whereas nanodiscs restricted the transporter in the IF-narrow conformation. Some detergents like LMNG allowed both IF-wide and IF-narrow conformations.

In contrast to a single, wide-open conformation of MsbA in DDM, DrAbcb4 shows multiple IF conformations in DDM; for example, five IF conformations were observed for DrAbcb4 in the apo state, suggesting that DrAbcb4 in DDM retains its conformational flexibility, compared to the prior structures obtained with restrictions imposed by antibodies, crystal packing, or nanodiscs. However, as shown in the MsbA paper, the choice of detergents and lipids may bias certain conformations, which suggest the possibility that the use of different detergents or drugs may cause shifts in the distribution of the conformational landscape of DrAbcb4 as shown in Figure 1 below (Supplementary Figure 8A in the revision).

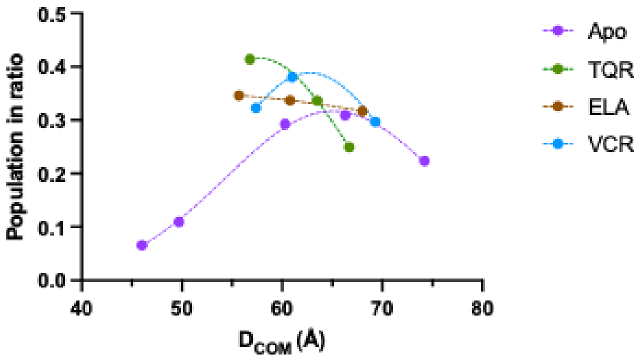

Figure 1. DrAbcb4 conformational landscape depicted as the normalized particle numbers as a function of D_{COM} can be modulated by presence of substrate/inhibitor.

We also attempted cryo-EM structure determination of DrAbcb4 reconstituted in nanodiscs using MSP1D1. Only low-resolution IF-narrow structures were obtained, from which we could measure separations of NBDs and conclude that DrAbcb4 in nanodiscs has its conformation restricted (Figure 2), as in the case of MsbA in nanodiscs.

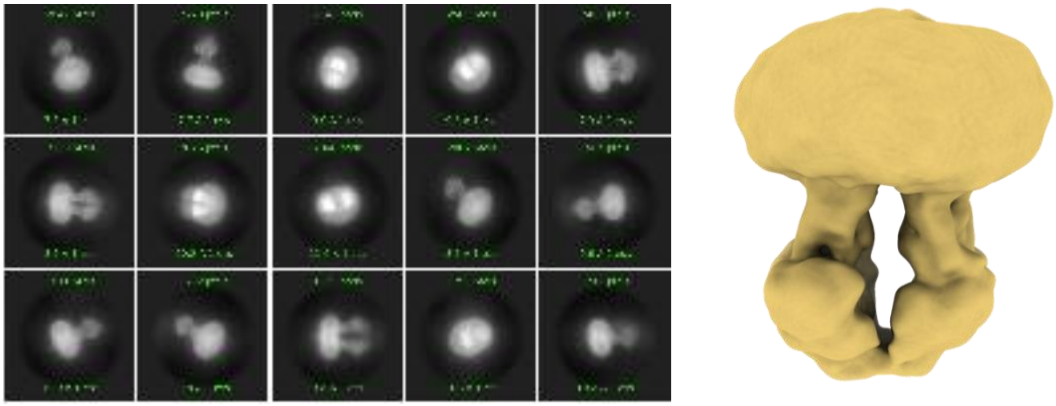

Figure 2. Cryo-EM analysis of DrAbcb4 reconstituted into nanodisc. Left panel: 2D classification. Right panel: representative 3D reconstruction.

In the revision, we changed the sentence in the Results section on page 8 from "The overall separations (D_{COM}) observed in the presence of inhibitor and substrate were consistently narrower compared to those of the apo dataset (Table 1, Figure 1C), indicating that drug-binding may induce closing of the two lobes." to "The overall conformational landscape of DrAbcb4, which is represented by the distribution of normalized particle numbers going into the structures with different D_{COM} values, was consistently shifted towards narrower separations between the two lobes in the presence of drugs compared to the apo dataset (Supplementary Figure 8A), indicating that drug-binding may affect conformational equilibrium."

Also, in the Discussion on Page 16, a paragraph was added "Recent cryo-EM reconstructions of MsbA demonstrated that the IF conformation of the transporter, either open wide or narrow, is modulated by detergent or nanodisc[40]. Our study of DrAbcb4 embedded in nanodisc also produced narrowly open IF structure, albeit at low resolution (Data not shown). By contrast, the presence of multiple IF conformations of apo DrAbcb4 indicates that detergents like DDM do not restrict DrAbcb4 conformational flexibility (Fig. 1). However, we cannot exclude the possibility that different detergents may alter the distribution of the conformational landscape of DrAbcb4, as observed for the drug-bound samples (Fig 2, Supplementary Figure 8A)."

Comment (9): The authors describe conformational adaptations and suggest that rather than the transport substrate binding affecting the NBD distance, nucleotide binding affects the NBD

distance. The authors conclude that the various conformations serve as a versatile binding site, explaining poly specificity. If this were true, the authors need to show that all these conformations exist in the apo state. This would certainly deserve a separate figure.

Response: To clarify, we did not say "nucleotide binding affects the NBD distance". We emphasize that our data are consistent with (1) DrAbcb4 undergoes spontaneous open-and-close movement in resting state in the presence of nucleotide. (2) This open-and-close movement is coupled to individual helix dynamics, including rotation, tilting, writhing, etc. (3) Movement of helices accompanies changes in substrate-interacting surface, permitting priming of substrates for binding. (4) As a result, the interaction patterns between P-gp and its substrates go far beyond the conventional one-on-one enzyme-substrate interactions defined by the lock-and-key mechanism.

Our dynamic sampling hypothesis is supported by existing structural data. For example, a single P-gp conformation can bind different compounds or multiple of the same compound. More importantly, compounds can bind to P-gp in different poses and in different protein conformations.

Although limited resources bar experimental determination of all possible conformations existed in the apo state as suggested, more conformations have been obtained over time. There are now 69 entries in the PDB of structures of P-gp and its homologs, many of which are in the apo state. Our hypothesis can also be further tested through identification of new conformations in the future when more conditions can be experimented.

Comment (10): In general, the authors need to be more specific when describing the conformations. Their data is rich and insufficient to address conformational changes globally, but they must investigate the underlying shift in detail.

Response: In the revision, we try to emphasize the definitions of DrAbcb4 conformational changes more clearly and consistently. We define global conformations as changes in the overall shape of the DrAbcb4 molecule, starting from various IF conformations such as IF-wide, IF-med, IF-narrow, to OF. We follow the conventions in the field to designate specific conformations such as IF-occluded and IF-closed. We use helix rotation, tilting, unwind, positional shifts, etc. for motions accompanying global conformational changes. The global motion can be seen as a culmination of many local movements, resembling numerous small moves by cogs and gears to make a clock tick.

For example, to describe the underlying helix shift in detail, we added 3 panels in the revised Supplementary Figure 8 and modified the Results and Discussion sections accordingly. Please refer to our response to Comments (4) and (5) above.

Minor Comments

Comment (11): It would serve readability if the authors were more specific in some sections of the

manuscript. For example, in line 264 “We noticed that in drug-bound structures such as DrAcb4/TQR, some helices display large and <HTA> values, deviating significantly from trending lines, indicating involvement of residues in these helices in drug binding.” I would immediately like to know which helices are being discussed, and I do not think that this is adding to clarity.

Response: Structural changes can be detected by various comparative analysis methods. Drug-bound structures were found to be outliers in the ΔD_{COM}-RMSD analysis presented in Fig 2B and Supplementary Figure 8B and were labelled out in the revision. Large numbers off trendlines in HRA and HTA analyses are likely due to helix repacking or drug binding. These methods can also detect likely deviations present in some pdb files. In the revision, we modified this sentence to “We noticed that some helices display <HRA> and <HTA> values that deviate significantly from trend lines, suggesting possible roles in drug binding or in helix repacking associated with particular conformational states”.

Comment (12): I do not think that the authors show this:
Line 380: In this study, the similarity extends beyond function and structure to include the mechanism of function, as revealed by the structures of DrAbcb4, both drug-free and drug-bound, in the presence of ATPS/Mg2+

Response: We meant to say, "In this study, the similarity extends beyond function and sequence to include the structure and mechanism of function". This is the point we want to make – shared mechanism in addition to sequence and structural similarity. This sentence has been revised.

Comment (13): Line 43: ATP hydrolysis by the NBDs drives conformational changes of the TMDs, enabling substrate binding and release on opposite sides of the membrane. This statement is incorrect, as it has been shown several times that already ATP binding drives the conformational change.

Response: The sentence is changed to "ATP binding and hydrolysis by the NBDs couple to conformational changes of the TMDs ..."

Comment (14): Figure1 and 2 – should be improved for clarity. The structures and models are tiny, and so is the text. Neither is easy to understand.

Response: In the revision, we revised Figure 1 and 2 for better clarity.

Reviewer #2 (Remarks to the Author):

comments:

Comment (1): This is a nice manuscript revealing unconstrained cryo-EM structures of multiple zebrafish abcb4 (unlike the many constrained structures of human ABCB1), an ortholog of human ABCB 1that appears to be for the most part functionally similar to human ABCB1 in that abcb4 is localized to the zebrafish blood-brain barrier. With respect to orthologs, there are differences among rodent, human and zebrafish P-gps in that their linkers are of different length. This seems important because length of the linker affects both basal and substrate stimulated activity. In this respect it would be nice if the authors could address if this (or other factors) account for differences between abcb4 and abcb1 with respect to verapamil stimulation and inhibition by cyclosporin. Do differences in linker account for this or are there other factors?

Response: We appreciate the reviewer's positive comments on our findings. With respect to the relationship between linker lengths and ATPase activities, we are not aware of any study that compares ATPase activities of orthologs with different linker lengths. Conceivably, ATPase activity is affected by many factors including the lipid environment (soy lipid used in this work), choice of assay buffer and detergents, and assay temperature used. In the literature, the ATPase activities of P-gp were reported under many different conditions and in wide ranges. The importance of assay conditions on ATPase activity can be appreciated for the bacterial ABC transporter MsbA that shows higher ATPase activity when reconstituted into nanodics compared to those measured in detergents [13].

In the case of comparing mP-gp, hP-gp, and DrAbcb4, their linker lengths are 77, 77, and 71 amino acid residues, respectively, and their ATPase activities are comparable. ATPase activities we reported in this work for DrAbcb4 were carried out at 30°C, whereas most ATPase activity reported for mouse and human P-gp were measured at 37°C.

While the linker in all structures of P-gp determined to date is disordered, its functional importance is indisputable. Hrycyna et al. [14], reported major deletion (34 residues) or sequence alterations of the linker of hP-gp led to changes in ATPase activity and abolishing of transport activity and verapamil-stimulated ATPase activity. This being said, the minimal shortening of the unstructured DrAbcb4 linker compared to mouse and human seems unlikely to affect our interpretation of these results.

Comment (2): Human Abcb1 when comparing the apo to substrate-bound structure show large, asymmetric rearrangements within the membrane. Some of these changes are thought to have a gating function, perhaps through effects on an autoinhibitory TM? It would be helpful to know if similar changes and analogous domains are operative in abcb4. If not, please account for why not.

Response: We think that the reviewer here refers to a recent publication by Kurre et al [6] reporting structures of hP-gp reconstituted into saposin nanodiscs and in the absence/presence of bound drugs (PDBs: 9CR8, 9CTC, 9CTF, 9CTG). Three of these structures have relatively small NBD separations. 9CTG is in the OF conformation and 9CTC is in the IF-occluded conformation. Both conformations were previously reported. 9CR8 is an apo structure also having a small NBD separation and designated as an IF-closed conformation, because it is similar to the IF-occluded conformation but has the drug-binding cavity

collapsed by further kinking of TMH4. The authors speculated that this conformation (IF-closed) is "auto-inhibitory". One additional structure, 9CTF, with bound taxol displays a large NBD separation of 58.8 Å.

In our study of DrAbcb4, we did not observe any measurable populations via classification that led to OF, IF-occluded, or IF-closed conformations. Instead, most of our DrAbcb4 structures in the IF conformations have wider NBD separations than that of 9CTF up to 75 Å. 9CTF has a D_{COM} of 58.8 Å that is very similar to VCR-IF^{Nar}, which is DrAbcb4 in the IF conformation with bound vincristine and a D_{COM} of 57.6 Å. Importantly, neither VCR-IF^{Nar} nor 9CTF shows TMH4 kinking.

The three structures of hP-gp with very small D_{COM} all display TMH4 kinking. Interestingly, the structures of DrAbcb4 with larger NBD separations also show TMH4 kinking but in the opposite direction to the ones reported in the EMBO J paper, as shown in Figure 3C of our submitted paper.

In the revision, we added a Supplementary Figure 8D (Figure 3 below) to show the conformations of TMH4 and TMH10 at different D_{COM} values. We revised the Results on page 10 as follows: "The movement of TMHs 4 and 10 of DrAbcb4 drives the SBP wide open, in stark contrast to the enclosed SBP induced by kinking of TMHs 4 and 10 observed in P-gp's IF-Occluded state (Supplementary Figure 8D). The latter led to the proposed substrate gating mechanism[18]". In the Discussion, we also added the following sentence (on page 20, in bold): "One question arises as to how P-gp prevents the bound substrates from escaping its binding cavity? A likely solution is through the previously described "IF-Occluded state" (Fig. 5), **in which the SBP is enclosed from the cytosol by kinked TMHs 4 and 10[30,44]. Although we have not yet captured DrAbcb4 in the IF-Occluded state in our current study, it's likely that DrAbcb4 would share a similar gating mechanism**".

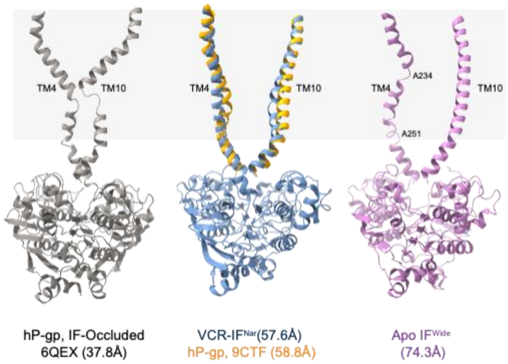

Figure 3. Conformation of TMHs 4 and 10 at different D_{COM} values. For clarity, the rest of TMHs are not shown. This figure is incorporated as Supplementary Figures 8D in the revision.

In conclusion, we believe that it is possible when DrAbcb4 were in the conformations comparable to hP-gp (D_{COM} is at about 38 Å or closer), TMHs 4 and 10 would adopt the kinked conformation as a gating mechanism as observed in hP-gp.

Comment (3): Additionally, some determinations of how the size and volume of the binding pocket change would be important to include and comment if this is related to packing of the TMD.

Response: Following the reviewer's suggestion, we performed volume measurement using KVFinder, which is one of frequently used programs in determining surface property and volume of substrate-binding pockets (SBP) in proteins. We also investigated the potential connection between changes in the SBP volume and helix repacking during the open-and-close movement of the DrAbcb4 two lobes.

Helix repacking for P-gp was first proposed from an early EM study of 2D-crystals at low resolution under the nucleotide treated and untreated samples, where EM densities for TMHs were shown to be shifted in the two samples [15]. Molecular modeling and trajectory analysis of MD simulations of human P-gp and its mutants also indicated shifting of TMH positions as well as changes in volume distribution [16]. In our structural study of DrAbcb4, the observed open-and-close movement of the two lobes expressed as D_{COM} correlates with individual helix's movement that includes rotation, tilting, and writhing etc. Although relative helix rotation and tilting are often linear within a given range of D_{COM}, dramatic changes in helix rotation and tilting were observed at certain D_{COM} values, for example when D_{COM} becomes very large as shown in Figures 3E and S9. Helix repacking takes place when the transporter is undergoing transition from IF to OF or when the two lobes either approaches each other very closely or extends very large due to, for example, partial unwinding, kinking, and position-shifting of TMH4 and TMH10 (Figure 3B and 3C).

In the revision, we added a Supplementary Figure 8C (Figure 4 below) to illustrate large movement of helices or helix repacking, which occurs at very large D_{COM}.

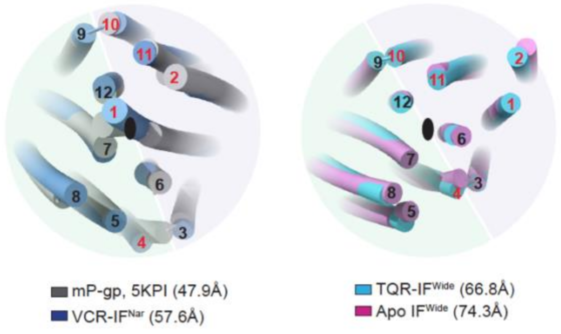

Figure 4. Top view of the TMDs from the extracellular side, with the TMD1 shaded in light grey and TMD2 shaded in light green. Comparing structures with small D_{COM} of 47.9Å and 57.6 Å (Left) to those with large D_{COM} of 66.8 Å and 74.3 Å (Right), several TMHs shift places indicating helix repacking. The pseudo-2-fold axis is shown as black oval. This figure is incorporated in the revision as Supplementary Figure 8C.

To investigate how the volume of the SBP relates to changes in D_{COM}, we first must define the boundary for SBP, which we decided to include only the TM region of the structure (Figure 5 below). Using a probe of 3 Å radius, we measured the SBP volumes for those ligand-free DrAbcb4 structures (Table 1 below).

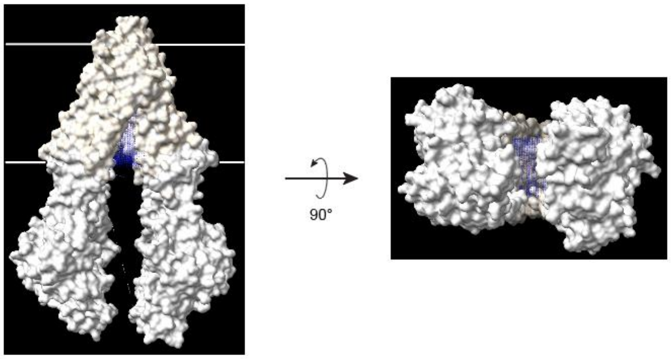

Figure 5. Definition of substrate-binding pocket (SBP) of DrAbcb4. Only the portion of the structure within the boundary of membrane represented by the two parallel lines were used. Here the structure is represented by a molecular surface in gray and grid points representing SBP volume.

It was noticed that the volume of SBP increases rapidly as a function of D_{COM} and reaches a plateau when the D_{COM} is in the range of 66-68Å. Further increase in the D_{COM} value (> 68Å) leads to helix repacking and a modest increase in SBP volume. It should be pointed out that the volume of SBP is affected by the probe radius and binding of ligands also changes the volume (data not shown).

Table 1. Relationship of D_{COM} with the estimated volume of the substrate-binding pocket*.

	APO-IF^{Nar}	VCR-IF^{Med}	Apo-IF^{Med}	TQR-IF^{Wide}	ELA-IF^{Wide}	VCR-IF^{Wide}	Apo-IF^{Wide}
D_{COM} (Å)	60.3	61.0	66.3	66.7	68.0	69.3	74.2
Vol (Å^3)	1,427	2,224	3,466	3,226	3,425	3,857	3,861

*A probe radius of 3 Å was used.

In the revision, we added sentences in the Results section on page 10 "(5) The volume of the SBP encompassing the membrane part of DrAbcb4 increases with D_{COM}, before gradually plateauing once D_{COM} reaches approximately 66Å or wider", and "Observed positional shifts of helices as the two lobes of DrAbcb4 open wider involve re-arrangement of TMHs in the TMDs (Supplementary Figure 8C), which resembles helix repacking previously described for hP-gp undergoing IF to OF transition[32]. Helix repacking also leads to abrupt changes in the volume of the SBP. Conceivably, repacking and unwinding are ... ".

Comment (4): Elacridar shows inhibition whereas tariquidar appears not to. While the authors propose helix rotation and tilt as important features associated with ligand binding it is hard to reconcile these disparate effects. Are there dramatic differences in interaction with key amino acid residues for these two inhibitors? Does tariquidar actually behave more like a substrate for Abcb4 and are there marked differences in the contact frequency of key residues? It appears that elacridar affects on the NBD separation is more similar to vincristine than to tariquidar. Thoughts on this?

Response: The reviewer is referring to Figure S1C showing ATPase activities as a function of vincristine, tariquidar, and elacridar concentrations. In the paper, we used four compounds: verapamil, tariquidar, elacridar, and vincristine. While verapamil gave a classic bell-shaped ATPase activity profile for a P-gp inhibitor (Fig. S1B), those for vincristine, tariquidar, and elacridar deviate from it quite significantly. Among the four compounds, only vincristine is a substrate, the other three are inhibitors for DrAbcb4 [1]. As the reviewer correctly pointed out, tariquidar and elacridar displayed disparate ATPase profiles, while structural explanation underlying these observations is lacking. Shukla et al reported P-gp inhibitors such as zosuquidar stimulated the ATPase rate of detergent (DDM)-solubilized ABCB1 but inhibited the lipid-reconstituted ABCB1 ATPase rate [17]. This opposing effect could not be explained by the structures of zosuquidar-bound P-gp isolated in detergent or in lipid, as the binding interactions of the zosuquidar molecules identified in the two conditions are similar [18]. Compounding these difficulties, structural data could not explain the phenomenon of "basal" ATPase activity, which is the ATPase activity in the absence of added drugs, because in all the structures we determined (all DrAbcb4 apo conformations) as well as those by others in the absence of added drugs (human 8GMG; mouse 5KPI and 4Q9H; C. elegance 4F4C), frequently additional EM densities in the SBP can be found.

From a structural perspective, we observed (1) tariquidar and elacridar have different binding modes. Either one or two copies of tariquidar can bind to the TMD, whereas only one copy of elacridar was found in the binding site (Figure 4B and 4C). (2) Different sets of residues were identified interacting with tariquidar and elacridar (Figure 4D). For example, L344 is used to contact tariquidar, it is not used by elacridar. Despite these differences, it is difficult to pinpoint exactly how they lead to differences in ATPase activities. In order to fully understand the determinants that dictate the rate of ATPase activity, it will require more high-resolution structures with different bound compounds coupling to biochemical and biophysical measurements. It will be a joint effort in the field.

References

1. Robey RW, Robinson AN, Ali-Rahmani F, Huff LM, Lusvarghi S, Vahedi S, et al. Characterization and tissue localization of zebrafish homologs of the human ABCB1 multidrug transporter. Sci Rep. 2021;11(1):24150. Epub 2021/12/19. doi: 10.1038/s41598-021-03500-8. PubMed PMID: 820; PubMed Central PMCID: PMCPMC8683423.
2. Park J, Kim H, Alabdalla L, Mishra S, McHaourab H. Generation and characterization of a zebrafish knockout model of abcb4, a homolog of the human multidrug efflux transporter P-glycoprotein. Hum Genomics. 2023;17(1):84. Epub 20230906. doi: 10.1186/s40246-023-00530-3. PubMed PMID: 880; PubMed Central PMCID: PMCPMC10481557.
3. Patton EE, Zon LI, Langenau DM. Zebrafish disease models in drug discovery: from preclinical modelling to clinical trials. Nat Rev Drug Discov. 2021;20(8):611-28. Epub 20210611. doi: 10.1038/s41573-021-00210-8. PubMed PMID: 907; PubMed Central PMCID: PMCPMC9210578.
4. Young MA, Rees SD, Nicklisch SCT, Stowell MHB, Hamdoun A, Chang G. Cryo-EM Structures of Apo and DDT-Bound P-Glycoprotein in Yellowfin Tuna. Environ Sci Technol. 2025. Epub 20250529. doi: 10.1021/acs.est.5c03398. PubMed PMID: 892.
5. Shabbir A, DiStasio S, Zhao J, Cardozo CP, Wolff MS, Caplan AJ. Differential effects of the organochlorine pesticide DDT and its metabolite p,p'-DDE on p-glycoprotein activity and expression. Toxicol Appl Pharmacol. 2005;203(2):91-8. doi: 10.1016/j.taap.2004.07.011. PubMed PMID: 909.
6. Kurre D, Dang PX, Le LTM, Gadkari VV, Alam A. Structural insights into binding-site access and ligand recognition by human ABCB1. EMBO J. 2025. Epub 20250113. doi: 10.1038/s44318-025-00361-z. PubMed PMID: 876.
7. Schaefer KG, Roberts AG, King GM. Advantages and potential limitations of applying AFM kymograph analysis to pharmaceutically relevant membrane proteins in lipid bilayers. Sci Rep. 2023;13(1):11427. Epub 20230715. doi: 10.1038/s41598-023-37910-7. PubMed PMID: 886; PubMed Central PMCID: PMCPMC10349840.
8. Nguyen PH, Sigdel KP, Schaefer KG, Mensah GAK, King GM, Roberts AG. The effects of anthracycline drugs on the conformational distribution of mouse P-glycoprotein explains their transport rate differences. Biochemical pharmacology. 2020;174:113813. Epub 20200116. doi: 10.1016/j.bcp.2020.113813. PubMed PMID: 887; PubMed Central PMCID: PMCPMC8262137.
9. Galazzo L, Meier G, Januliene D, Parey K, De Vecchis D, Striednig B, et al. The ABC transporter MsbA adopts the wide inward-open conformation in E. coli cells. Sci Adv. 2022;8(41):eabn6845. Epub 20221012. doi: 10.1126/sciadv.abn6845. PubMed PMID: 867; PubMed Central PMCID: PMCPMC9555771.
10. Hamaguchi-Suzuki N, Adachi N, Moriya T, Yasuda S, Kawasaki M, Suzuki K, et al. Cryo-EM structure of P-glycoprotein bound to triple elacridar inhibitor molecules. Biochemical and biophysical research communications. 2024;709:149855. Epub 20240328. doi: 10.1016/j.bbrc.2024.149855. PubMed PMID: 850.
11. Alam A, Kowal J, Broude E, Roninson I, Locher KP. Structural insight into substrate and inhibitor discrimination by human P-glycoprotein. Science. 2019;363(6428):753-6. Epub 2019/02/16. doi: 10.1126/science.aav7102. PubMed PMID: 718.
12. Verhalen B, Ernst S, Borsch M, Wilkens S. Dynamic ligand-induced conformational rearrangements in P-glycoprotein as probed by fluorescence resonance energy transfer spectroscopy. J Biol Chem. 2012;287(2):1112-27. Epub 20111115. doi: 10.1074/jbc.M111.301192. PubMed PMID: 906; PubMed Central PMCID: PMCPMC3256922.
13. Hoffmann L, Baier A, Jorde L, Kamel M, Schafer JH, Schnelle K, et al. The ABC transporter MsbA in a dozen environments. Structure. 2025;33(5):916-23 e4. Epub 20250307. doi: 10.1016/j.str.2025.02.002. PubMed PMID: 904.

14. Hrycyna CA, Airan LE, Germann UA, Ambudkar SV, Pastan I, Gottesman MM. Structural flexibility of the linker region of human P-glycoprotein permits ATP hydrolysis and drug transport. Biochemistry. 1998;37(39):13660-73. PubMed PMID: 232.
15. Rosenberg MF, Velarde G, Martin C, Berridge G, Kerr ID, Callaghan R, et al. Repacking of the transmembrane domains of P-glycoprotein during the transport ATPase cycle. European Molecular Biology Organization Journal. 2001;20:5615-25. PubMed PMID: 4.
16. Bonito CA, Ferreira RJ, Ferreira MU, Gillet JP, Cordeiro M, Dos Santos D. Theoretical insights on helix repacking as the origin of P-glycoprotein promiscuity. Sci Rep. 2020;10(1):9823. Epub 20200617. doi: 10.1038/s41598-020-66587-5. PubMed PMID: 908; PubMed Central PMCID: PMCPMC7300024.
17. Shukla S, Abel B, Chufan EE, Ambudkar SV. Effects of a detergent micelle environment on P-glycoprotein (ABCB1)-ligand interactions. J Biol Chem. 2017;292(17):7066-76. doi: 10.1074/jbc.M116.771634. PubMed PMID: 662; PubMed Central PMCID: PMCPMC5409473.
18. Alam A, Kung R, Kowal J, McLeod RA, Tremp N, Broude EV, et al. Structure of a zosuquidar and UIC2-bound human-mouse chimeric ABCB1. Proc Natl Acad Sci U S A. 2018;115(9):E1973-E82. Epub 2018/02/15. doi: 10.1073/pnas.1717044115. PubMed PMID: 684; PubMed Central PMCID: PMCPMC5834697.

REVIEWER COMMENTS

Reviewer #1 (Remarks to the Author):

The authors made a solid effort and addressed almost all of my comments. However, aside from an attempt to reconstitute DrAbcb4 into a nanodisc, the authors largely refrained from performing the required additional experiments. For many of my comments, this is acceptable, but I am surprised that my comment 7 is not properly addressed. I think I opened an avenue to experimentally validate this claim, even suggesting a simple processing-based experiment that could have been performed without collecting additional data or generating new samples.
The literature is full of claims where lower achievable resolution in a single cryo-EM experiment is equated with greater flexibility. This can obviously be true, but often wrong. It has to be validated! The vinvristine dataset contains a third of the raw micrographs and a third of the particles in each map. Thus, lower resolution is to be expected - regardless of flexibility.
I suggested a facile experiment that could directly address this claim and validate it (or not). I am puzzled by the authors' preference to evade this obvious solution to their experimental data and instead rephrase the text.

Previous comments by the Reviewer 1 on original version:
Comment (7): The authors suggest that two of the tested drugs (elacridar and tariquidar) stabilize the IF conformation, whereas vincristine destabilizes the protein. They base this claim on the different resolutions that they were able to obtain in the cryo-EM reconstructions. This is an interesting finding, but it also demands a careful statistical analysis. In general, it is not easy to get statistics between different cryo-EM datasets as numerous factors such as ice thickness, particle concentration and orientation, CTF-values, and number of particles in the final densities, all together have a significant impact on the achievable resolution. In analogy to biochemical experiments, biological replicates would be required to validate such an interpretation. As cryo-EM data collection is expensive and time-consuming, the acquisition of biological replicates may not always be possible; therefore, it is essential to mine the dataset as much as possible to consolidate the findings. An acceptable option could be the generation of datasets into comparable groups across the obtained datasets. Through this, at least CTF-values and particle numbers, etc., can be homogenized, which would provide a more reliable statistic.

Response: We appreciate the reviewer's interest in whether the observed difference in resolution between our reconstructions reflects disparities in dataset size and quality, rather than conformational flexibility.

In our initial submission, we stated that "Interestingly, reconstructions of the DrAbcb4/TQR and DrAbcb4/ELA datasets reached higher resolutions than the drug-free DrAbcb4, while those from the DrAbcb4/VCR dataset had relatively lower resolutions (Supplementary Table 1), which suggests a potential stabilizing role of inhibitors, but not substrates, to the conformations of DrAbcb4 in solution". This statement was questioned by the Review 1, who pointed out that the achievable resolution of a reconstruction is influenced by many factors. In the previous revision, we rephrased the text to "Reconstructions from the DrAbcb4/TQR and DrAbcb4/ELA datasets reached higher resolution than the

drug-free DrAbcb4, while those from the DrAbcb4/VCR dataset had relatively lower resolution (Supplementary Table 1)."

This change reflects our agreement with the reviewer that the achievable resolution of a cryo-EM reconstruction is influenced by multiple factors, both apparent and latent, that are difficult to standardize across independent datasets. Consequently, it is insufficient to rely on resolution alone as evidence of a stabilization effect by inhibitors.

In this current revision, we have conducted the processing-based control analysis as suggested by the reviewer.

To minimize dataset heterogeneity, we first performed the "Manually Curate Micrographs" job in CryoSPARC for each dataset, applying consistent criteria based on defocus range, relative ice thickness, and CTF fit resolution. Using the curated (homogenized) micrographs with CTF fits better than 7 Å, we then randomly selected 98,961 particles from each remaining particle stack and carried out non-uniform refinement, and the results are summarized in the table and figure below.

Table. Comparison of "Achievable Resolution" between full data set and that with randomly selected 98,961 particles. Our result is also compared to published work.

Data set name	Original full dataset			98,961 particles randomly selected from homogenized micrographs
	PDB	Particle number	Resolution (Å)	Resolution (Å)
IF^{Nar}	9ODY	180,825	3.29	3.56
IF^{Med}	9OE0	190,766	3.39	3.59
IF^{Wide}	9ODZ	138,295	3.59	3.70
VCR-IF^{Nar}	9OE7	110,004	3.95	3.97
VCR-IF^{Med}	9OE8	129,591	3.70	3.82
VCR- IF^{Wide}	9OE9	101,031	4.09	4.10
TQR-IF^{Nar}	9OE1	619,997	3.15	3.34
TQR-IF^{Med}	9OE2	504,014	3.16	3.37
TQR- IF^{Wide}	9OE3	373,818	3.53	3.68
ELA -IF^{Nar}	9OE4	582,777	3.09	3.46
ELA -IF^{Med}	9OE5	567,429	3.26	3.55
ELA- IF^{Wide}	9OE6	533,945	3.32	3.59
hP-gp/MRK16	7A65	115,341	3.9	
hP-gp/MRK16/Vincristine[1]	7A69	160,170	3.2	
hP-gp/MRK16/Elacridar[1]	7A6C	241,885	3.6	
hP-gp/MRK16/Tarquidar[1]	7A6E	160,295	3.6	
hP-gp/MRK16/Zosuquidar[1]	7A6F	166,803	3.5	

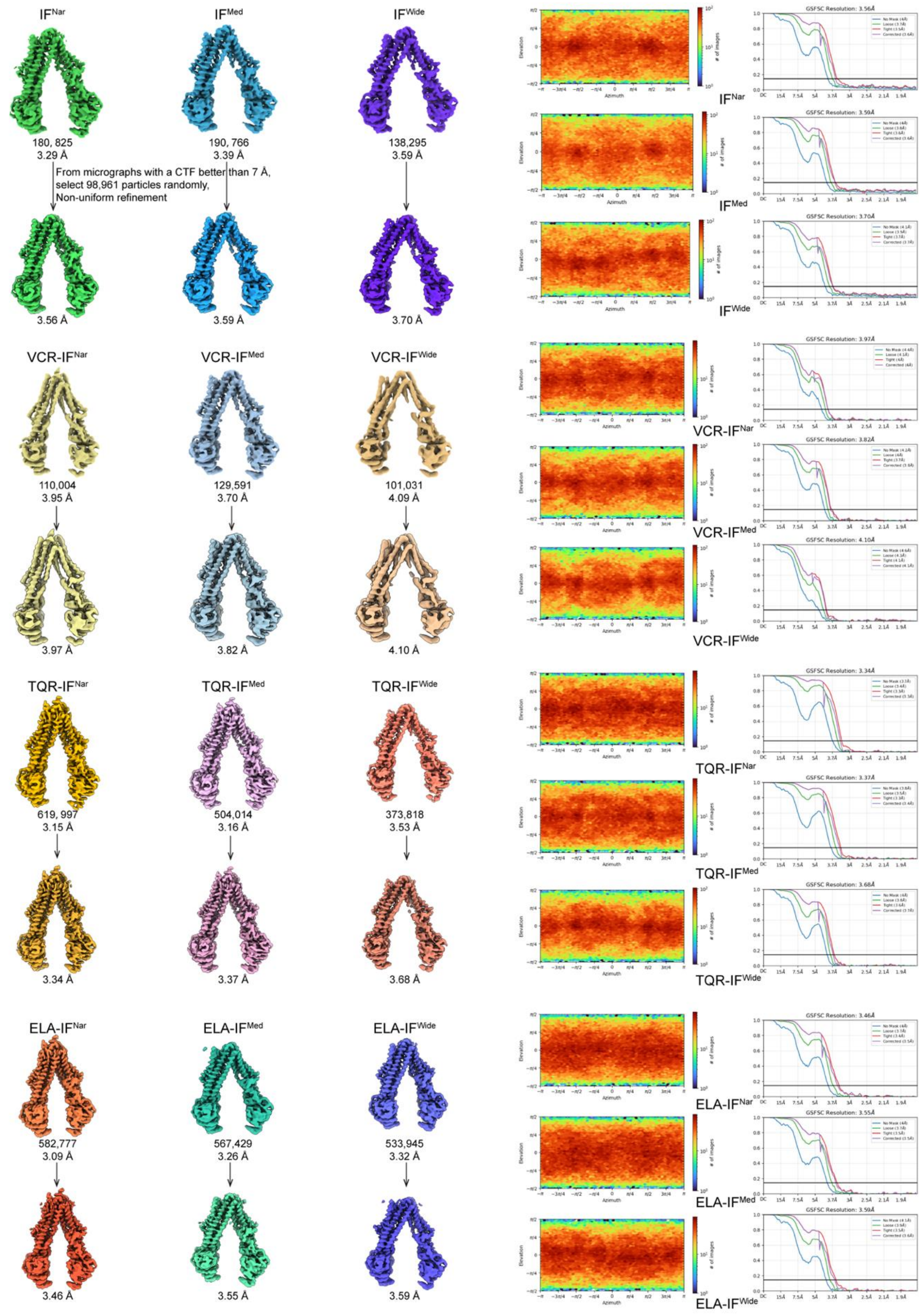

Figure. Data processing using fixed number of particles selected from homogenized raw micrographs

As shown in the table and figure, when the same number of particles was randomly selected from the homogenized micrographs, the reconstructions from the DrAbcb4/TQR and DrAbcb4/ELA datasets achieved slightly higher resolution compared to the apo dataset, whereas the reconstructions from the DrAbcb4/VCR dataset were of slightly lower resolution. Aside from the differences in reported resolution, the reconstructions are nearly identical with and without the normalization. Thus, the trend remains consistent with our original observation after unifying particle numbers and basic micrograph quality parameters, as the reviewer suggested.

However, we are not fully persuaded, as the reviewer previously noted, that the observed difference in reconstruction resolution can be attributed solely to the distinct effect of inhibitor versus substrate binding. Numerous experimental variables are inherently difficult to standardize across independent datasets. These include grid quality (e.g. bending and contaminations), particle distribution and preferred orientation, as well as microscope optical alignments, tuning, and session-specific data collection conditions.

Furthermore, we noted that Nosol, K. et al [1] reported in their 2020 PNAS paper that human P-gp structures in complex with the Fab fragment of MRK16 were determined in the apo, elacridar-, tariquidar-, zosuquidar- and vincristine-bound states. Notably, “the highest resolution was achieved for the vincristine-bound state of ABCB1–MRK16-Fab (3.2 Å)”. For completeness, the reported resolution and corresponding particle numbers for these structures are also included in the table for comparison.

Considering the modest differences observed in the homogenized datasets, the presence of the above-mentioned experimental variables that could not be fully controlled, and the contrasting observations reported in the literature[1], we believe it is more appropriate to refrain from concluding that inhibitor binding stabilizes the Abcb4 conformation.

Reviewer #2 (Remarks to the Author):

The authors have improved the manuscript. I have no additional amendments.

Response: We thank the reviewer for his/her positive assessment of our revised manuscript.

Reference

1. Nosol, K. et al. Cryo-EM structures reveal distinct mechanisms of inhibition of the human multidrug transporter ABCB1. Proc Natl Acad Sci U S A 117, 26245-26253 (2020).

Reviewer #1 (Remarks to the Author):

The authors now did the suggested control, and it turns out that their original claim was not substantial. They reverted this in the manuscript, and that's okay with me. I am a bit surprised about their wording, though, as they should be thankful to me for figuring this out. Too many papers in structural biology make claims without appropriate controls. It is very important that we, as a field, implement such necessary steps. Frankly, I am quite unhappy that it took two rounds of reviews before the authors performed this required, but simple, experiment. Once a claim is in the literature, it is very difficult to present something opposing, even if it is correct.

Response: We thank the reviewer for this important suggestion and appreciate his/her insight. We have revised the manuscript accordingly and believe these changes improve the rigor and clarity of our interpretation.
